# Efficacy and cosmetic outcomes of radiotherapy modalities in early-stage breast cancer: a systematic review and network meta-analysis

**DOI:** 10.3389/fonc.2026.1876930

**Published:** 2026-09-09

**Authors:** Yushan Dong, Tingting Liu, Peng Wang, Songjiang Liu

**Affiliations:** 1 Heilongjiang University of Chinese Medicine, Harbin, China; 2 The First Affiliated Hospital of Heilongjiang University of Chinese Medicine, Harbin, China

**Keywords:** accelerated partial breast irradiation, breast cancer, cosmetic outcomes, network meta-analysis, whole-breast irradiation

## Abstract

Breast cancer (BC) is a common malignancy with increasing annual incidence. Radiotherapy shows favorable clinical efficacy in BC management, but limited randomized controlled trial data is a major limitation. Therefore, further validation of the optimal radiotherapeutic strategy for early-stage BC is required. This network meta-analysis evaluated radiotherapy regimens’ effects on efficacy and cosmetic outcomes in early-stage BC, guiding clinical practice. A comprehensive literature search was conducted in PubMed, Cochrane Library, Embase, and Web of Science databases to identify randomized controlled trials and cohort studies investigating radiotherapy approaches for early-stage BC. Data screening and extraction were independently performed using a standardized protocol. Study quality was assessed using the Cochrane Risk of Bias tool and Newcastle–Ottawa Scale. The primary endpoint was ipsilateral breast recurrence (IBR), and secondary endpoints included locoregional recurrence, overall survival, disease-free survival, and cosmetic outcomes. Statistical analyses were performed using R 4.4.1 and STATA 18.0. A total of 36 studies involving 32,655 patients and 9 interventions were included. The lowest IBR rate was observed in patients treated with hypofractionated whole-breast irradiation (HF-WBI) (surface under the cumulative ranking curve [SUCRA], 10.54%), followed by conventional fractionated whole-breast irradiation (CF-WBI) (SUCRA, 35.70%). Compared with CF-WBI, intraoperative electron radiotherapy (IOeRT) (odds ratio [OR], 4.19; 95% credible interval [CrI], 1.83–10.26), intraoperative radiotherapy (IORT) (OR, 1.75; 95% CrI, 1.06–2.87), and HF-WBI (OR, 0.78; 95% CrI, 0.56–0.99) demonstrated statistically significant differences. The best cosmetic outcomes were achieved with three-dimensional conformal radiotherapy (3D-CRT) (SUCRA, 14.43%), followed by intensity-modulated radiotherapy (IMRT) (SUCRA, 16.04%). Compared with CF-WBI, 3D-CRT (OR, 0.11; 95% CrI, 0.01–0.85), IMRT (OR, 0.13; 95% CrI, 0.01–0.72), and IOeRT (OR, 0.25; 95% CrI, 0.04–0.78) showed statistically significant improvements. Since evidence supporting cosmetic outcomes is limited, findings should be interpreted with caution. Among patients with early-stage BC, HF-WBI was associated with the lowest rate of postoperative ipsilateral recurrence, whereas CF-WBI was associated with the second-lowest rate. In terms of cosmetic outcomes, 3D-CRT and IMRT demonstrated favorable clinical efficacy. Additional randomized controlled trials are necessary to clarify the comparative advantages and limitations of CF-WBI, HF-WBI, 3D-CRT, and IMRT, thereby strengthening the evidence base for early-stage BC radiotherapy strategies.

**Systematic review registration:**

https://www.crd.york.ac.uk/PROSPERO/, identifier CRD420251173161.

## 1 Introduction

Breast cancer (BC) remains the most common malignancy among women worldwide, and its incidence continues to increase annually. In 2020, approximately 2.3 million new cases were diagnosed globally (1). For patients with early-stage BC, the primary treatment objective is to achieve tumor eradication while preserving breast function and cosmetic appearance to the greatest extent possible. Currently, comprehensive treatment centered on surgery is considered the standard therapeutic strategy, and surgery combined with radiotherapy has become a major treatment modality for early-stage BC (2).

Radiotherapy is a cornerstone of BC management. In particular, adjuvant postoperative radiotherapy significantly reduces recurrence and mortality rates, thereby improving clinical outcomes and overall prognosis (3). Postoperative radiotherapy for BC primarily includes whole-breast irradiation (WBI) and accelerated partial breast irradiation (APBI). WBI protocols are categorized into conventional, hypofractionated, and ultra-hypofractionated regimens, whereas APBI includes several delivery modalities, including intraoperative radiotherapy (IORT), intraoperative electron radiotherapy (IOeRT), and external beam techniques such as intensity-modulated radiotherapy (IMRT) and three-dimensional conformal radiotherapy (3D-CRT). Collectively, these radiotherapy modalities provide patients with BC with a wider range of individualized treatment options (4).

Despite the diversity of available radiotherapy regimens and their unique advantages, comparative evaluation of their clinical efficacy remains a major focus of ongoing research. Although most primary studies and meta-analyses have mainly compared APBI with WBI, direct comparative evidence among different APBI techniques and various WBI fractionation schedules remains limited. Therefore, a systematic comparison of the efficacy of these radiotherapy approaches in patients with early-stage BC is of substantial clinical importance for treatment decision-making. Accordingly, in this study, we conducted a systematic review and network meta-analysis (NMA) to evaluate the comparative effectiveness of various WBI fractionation schedules and APBI modalities in early-stage BC, thereby providing robust evidence to support the selection of optimal radiotherapeutic strategies.

## 2 Materials and methods

This study was designed as a systematic review and NMA evaluating the clinical efficacy of different radiotherapy modalities for early-stage BC. The study design strictly followed the Preferred Reporting Items for Systematic Reviews and Meta-Analyses (PRISMA) guidelines, including the PRISMA extension statement for NMA of healthcare interventions (PRISMA-NMA) (5, 6). The study protocol has been registered in the International Prospective Register of Systematic Reviews (PROSPERO; #CRD420251173161).

### 2.1 Search strategy

A comprehensive literature search was performed in PubMed, the Cochrane Library, Embase, and Web of Science to identify studies published up to November 18, 2025. The search strategy included three major components: disease terms (“Breast Neoplasm,” “Breast Tumor,” “Breast Cancer,” “Breast Malignant Neoplasm,” “Breast Malignant Tumor,” “Mammary Cancer,” and “Breast Carcinoma”), whole-breast irradiation terms (“Whole-breast irradiation,” “whole breast irradiat,” “whole-breast radiotherapy,” and “WBI”), and accelerated partial breast irradiation terms (“Accelerated Partial Breast Irradiation,” “APBI,” “Interstitial Brachytherapy,” “Balloon-based Brachytherapy,” “IORT,” “Electron IORT,” “IMRT,” and “3D-CRT”). Detailed search strategies are presented in **Supplementary Data 1**.

### 2.2 Inclusion criteria

The inclusion criteria were established according to the Population, Intervention, Comparator, Outcomes, and Study design (PICOS) framework to ensure methodological rigor and comparability.

Population: Female Patients diagnosed with early-stage BC(Stage 0–IIIA) who had undergone breast-conserving surgery.

Intervention: Different APBI techniques, including IORT, IOeRT, IMRT, 3D-CRT, balloon-based brachytherapy, and interstitial brachytherapy, as well as hypofractionated and ultra-hypofractionated WBI.(It should be noted that, since both 3D-CRT and IMRT can be used for both WBI and APBI, it is clarified here that the studies included in this paper specifically refer to APBI based on 3D-CRT and IMRT technologies).

Comparator: Conventional fractionated WBI.

Outcomes: The primary outcome was ipsilateral breast recurrence (IBR). Secondary outcomes included locoregional recurrence (LR), overall survival (OS), disease-free survival (DFS), and cosmetic outcomes, as well as treatment-related Adverse Events.

Study design: Randomized controlled trials (RCTs) and cohort studies.

Language and Publication Status: As of November 18, 2025, full-text English articles published in peer-reviewed journals, with no restrictions on publication date.

### 2.3 Exclusion criteria

The exclusion criteria were as follows: (1) studies involving patients with non-early-stage BC or evaluating interventions and outcomes outside the predefined PICOS criteria; (2) non-primary literature, including reviews, meta-analyses, letters, and conference abstracts without complete methodological details; (3) studies with incomplete or non-extractable data; and (4) duplicate reports from the same trial or cohort, in which case the most comprehensive or most recent publication was included.

### 2.4 Literature screening and data extraction

After duplicate removal using EndNote 2025, two independent investigators (Y.S.D. and T.T.L.) screened titles and abstracts, followed by full-text evaluation of potentially eligible studies. Disagreements were resolved through discussion or consultation with a third reviewer (S.J.L.). Data extraction for the NMA was subsequently performed independently by the reviewers.

### 2.5 Data extraction and quality assessment

Data were extracted using a standard Excel spreadsheet. Extracted variables included author names, publication year, country, study design, intervention method, disease stage, sample size, IBR, LR, OS, DFS, and cosmetic outcomes. Two investigators (Y.S.D. and T.T.L) independently performed data abstraction; and discrepancies were resolved through consensus discussion.

For RCTs, the risk of bias was assessed using the Cochrane Risk of Bias tool, which evaluates seven domains: random sequence generation, allocation concealment, blinding of participants and investigators, blinding of outcome assessment, incomplete outcome data, selective reporting, and other potential sources of bias. Each domain was categorized as low, high, or unclear risk (7).

For cohort studies, methodological quality was evaluated using the Newcastle–Ottawa Scale (NOS), which assesses three dimensions: cohort selection, group comparability, and outcome assessment (8). Two investigators (Y.S.D. and T.T.L) independently assessed the methodological quality of the included studies, and discrepancies were resolved through consensus discussion.

### 2.6 Data analysis

The NMA was conducted using a Bayesian random-effects model to compare the efficacy of different intervention strategies. Statistical modeling was conducted using the Markov Chain Monte Carlo method, with four parallel chains. The first 20,000 iterations were used as the burn-in period, and the total number of simulation iterations was 50,000.

Model fit and global consistency were assessed using the deviance information criterion (DIC). In networks containing closed loops, node-splitting analysis was applied to evaluate local consistency. Furthermore, all treatment strategies were ranked according to their surface under the cumulative ranking curve (SUCRA) and league tables were generated to compare the relative efficacy of different interventions.

This study conducted separate analyses of RCTs and combined analyses of RCTs and cohort studies. All analyses were conducted in R version 4.4.1 (R Development Core Team, Vienna, http://www.R-project.org). For all outcomes, lower SUCRA values indicated better treatment effects.

## 3 Results

### 3.1 Characteristics of the included studies

**Figure 1:**
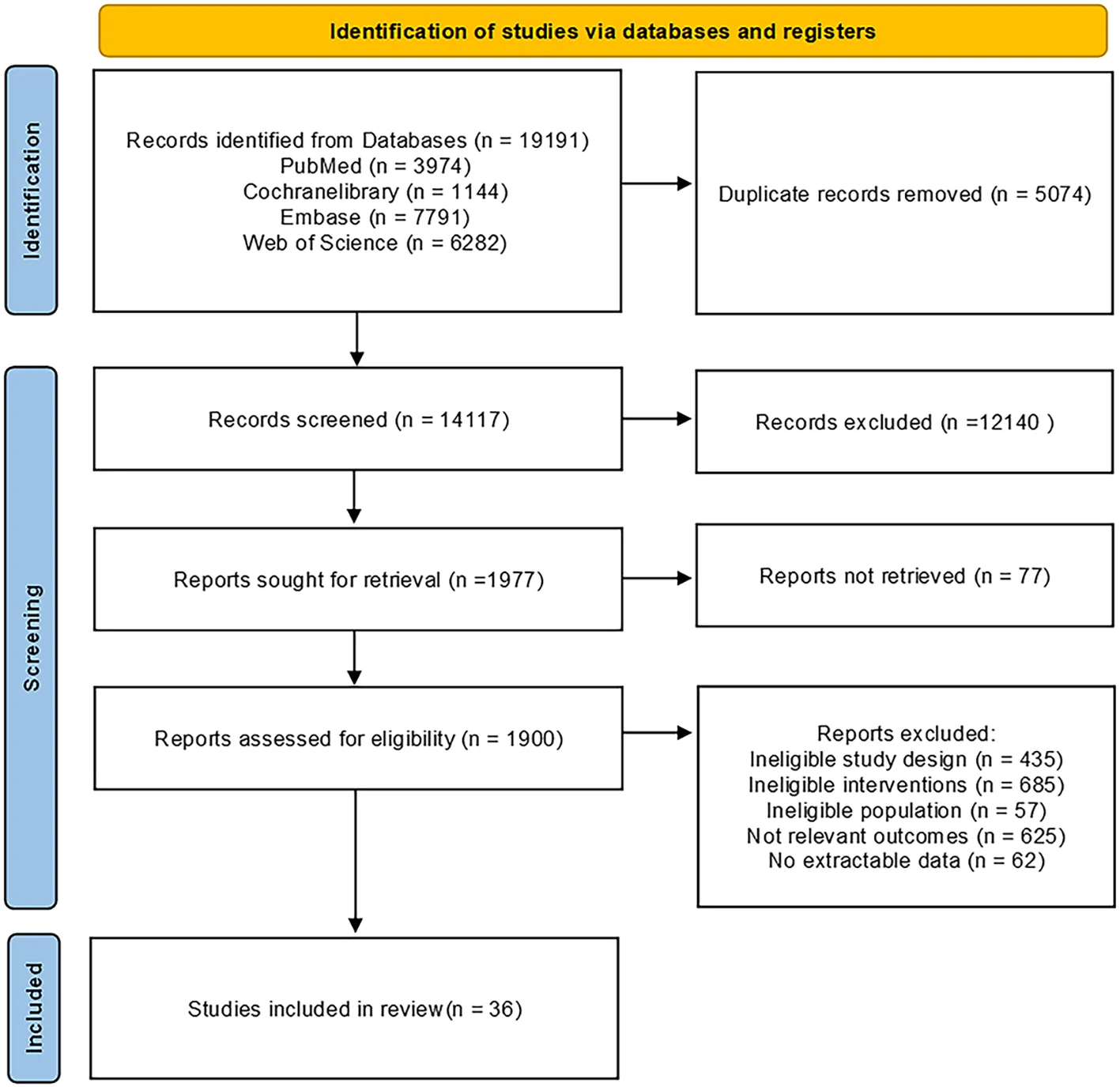
PRISMA flow diagram.

After removing duplicate records from the initial pool of 19,191 retrieved publications, 14,117 unique articles remained for screening. Subsequently, 12,140 articles were excluded, including animal studies (n = 63); reviews and meta-analyses (n = 1,707); conference abstracts, guidelines, case reports, and related publications (n = 4,533); and other ineligible studies (n = 5,837). As a result, we performed the full-text assessment of 1,977 articles. Ultimately, 36 studies were included in the final analysis (9–44), comprising 23 RCTs and 13 cohort studies. The literature selection process is presented in **Figure 1**.

The included studies showed no significant differences in baseline characteristics; all patients underwent breast-conserving surgery. The inclusion criteria restricted tumor staging to 0–IIIA, the rate of positive surgical margins was strictly controlled, and the hormone receptor-positive rate remained consistently high across studies, ensuring comparability. A comprehensive summary of the study characteristics is provided in **Table 1**.

**Table 1:** Characteristics of the included studies.

Study ID	Country	Research type	Treatment	Sample size	Age	Stage of BC	Hormone receptor, n (%)	Negative margin	Outcomes
Strnad et al.(2016) (9)	German	RCT	CF-WBI	551	62 (54–67)^*a^	0-IIA	HR+ 584 (93.0)	100%	IBR, OS, DFS, AE
			ISBT	633	62 (54–68)^*a^		HR+ 509 (93.0)	100%	
Livi et al. (2015) (10)	Italy	RCT	CF-WBI	260	<60 121 ≥60 139	0-IIIA	ER+ 249 (95.8) PR+ 235 (90.4)	NR	IBR, OS, cosmesis, AE
			IMRT	260	<60 102 ≥60 158		ER+ 248 (95.4) PR+ 232 (89.2)		
Meattini et al.(2020) (11)	Italy	RCT	IMRT	260	<60 102 ≥60 158	0-IIIA	ER+ 248 (95.4)	NR	IBR, LR, OS, AE
			CF-WBI	260	<60 121 ≥60 139		ER+ 249 (95.8)		
Polgár et al.(2013) (12)	Hungary	RCT	ISBT	128	59 (30-84)^*b^	I-IIA	ER+ 116 (90.6)	100%	IBR, cosmesis
			CF-WBI	130	58 (31-80)^*b^		ER+ 113 (86.9)	100%	
Polgár et al. (2007) (13)	Hungary	RCT	ISBT	128	59 (30-84)*^b^	I-IIA	ER+ 116 (90.6)	100%	IBR
			CF-WBI	130	58 (31-80)^*b^		ER+ 113 (86.9)	99.20%	
Yadav et al. (2020) (14)	India	RCT	3D-CRT	65	50 (32 – 75)^*b^	I-IIB	ER+ 38 (62.0) PR+ 23 (38.0)	92.30%	Cosmesis, AE
			HF-WBI	67	50 (27 – 67)^*b^		ER+ 39 (62.0) PR+ 24 (38.0)	89.60%	
Agrawal (2011) (15)	UK	RCT	CF-WBI	302	63.1 (50.0-88.4)^*b^	0-IIIA	NR	NR	IBR, OS, AE
			HF-WBI	308	62.9 (50.1-84.9)^*b^				
Rodrı´guez et al.(2013) (16)	Spain	RCT	CF-WBI	51	70.1 ± 5.2^*c^	I-IIA	ER+ 51 (100) PR+ 49 (96.1)	NR	cosmesis, AE
			3D-CRT	51	67.1 ± 6.1^*c^		ER+ 45 (88.2) PR+ 43 (84.3)		
Wang et al.(2020) (17)	China	RCT	CF-WBI	366	≥45 223	I-IIIA	ER+ 289 (79.4) PR+ 278 (76.4)	NR	IBR, LR, DFS, OS, cosmesis, AE
			HF-WBI	368	≥45 246		ER+ 300 (82.2) PR+ 297 (81.4)		
Veronesi et al.(2013) (18)	Italy	RCT	CF-WBI	654	<60 344 ≥60 310	0-IIIA	ER+ 589 (82.2) PR+ 512 (81.4)	NR	IBR, LR, OS, AE
			IOeRT	650	<60 321 ≥60 330		ER+ 583 (82.2) PR+ 487 (81.4)		
Ciabattoni et al.(2021) (19)	Italy	RCT	IOeRT	125	56.3 (29–75)^*b^	I-IIB	NR	NR	IBR, LR, cosmesis, AE
			CF-WBI	110	56.2 (34–75)^*b^				
Vaidya et al.(2020) (21)	UK	RCT	IORT	1140	<60 661 ≥60 479	I-IIB	ER+ 1005 (89.8) PR+ 1030 (91.7)	89.40%	IBR, OS
			CF-WBI	1158	<60 684 ≥60 474		ER+ 895 (80.3) PR+ 921 (82.7)	88.20%	
Polgár et al.(2017) (20)	Hungary	RCT	ISBT	633	62 (54–67)^*a^	0-IIA	HR+ 584 (93.0)	100%	cosmesis, AE
			CF-WBI	551	62 (54–68)^*a^		HR+ 509 (93.0)	100%	
Whelan et al.(2010) (22)	USA	RCT	CF-WBI	612	NR	I-IIA	NR	100%	IBR, OS, cosmesis, AE
			HF-WBI	622				100%	
Brunt et al.(2023) (23)	UK	RCT	CF-WBI	1361	60 (53–66)^*a^	I-IIIA	ER+ 1225 (90.0)	100%	IBR, LR, DFS, OS, AE
			HF-WBI	1367	61 (53–67)^*a^		ER+ 1244 (91.0)	100%	
Franceschini et al.(2021) (24)	Italy	RCT	HF-WBI	90	NR	I-IIB	ER+ 90 (100)	100%	IBR, LR, Cosmesis, AE
			IMRT	82			ER+ 82 (100)	100%	
Whelan et al.(2002) (25)	Canada	RCT	HF-WBI	622	<60 279 ≥60 340	I-IIA	ER+ 440 (71)	100%	IBR, DFS, OS, AE
			CF-WBI	612	<60 309 ≥60 303		ER+ 434 (71)	100%	
Vaidya et al.(2010) (29)	UK	RCT	IORT	1113	NR	I-IIB	NR	NR	IBR
			CF-WBI	1119					
Felice et al.(2017) (26)	Italy	RCT	CF-WBI	62	62 (39-82)^*a^	I-IIB	HR+ 50 (80.6)	NR	IBR, DFS, OS, AE
			HF-WBI	58	61 (40-81)^*a^		HR+ 52 (89.7)		
Vaidya et al.(2014) (27)	UK	RCT	IORT	1721	NR	I-IIB	NR	NR	IBR, LR, OS
			CF-WBI	1730					
Abo-Madyan et al.(2019) (28)	Germany	RCT	IORT	90	<60 29 ≥60 61	I-IIB	ER+77 (86) PR+ 73 (81)	NR	IBR, OS
			CF-WBI	90	<60 29 ≥60 61		ER+ 78 (87) PR+ 75 (83)		
Brunt et al.(2020) (30)	UK	RCT	CF-WBI	302	<60 112 ≥60 190	I-IIA	NR	100%	IBR, OS, AE
			HF-WBI	305	<60 110 ≥60 195			100%	
Shaitelman et al.(2018) (31)	USA	RCT	CF-WBI	149	<60 68 ≥60 81	0-IIB	ER+ 130 (87.2) PR+ 112 (75.2)	89.30%	IBR, cosmesis, AE
			HF-WB	138	<60 65 ≥60 73		ER+ 118 (85.5) PR+ 102 (73.9)	87.70%	
Chi et al.(2024) (32)	Taiwan,China	Cohort Study	IORT	112	<60 44 ≥60 68	0-IIA	ER+ 99 (88.3) PR+ 94 (83.9)	92.90%	LR, OS
			CF-WBI	135	<60 64 ≥60 71		ER+ 106 (78.5) PR+ 92 (68.1)	81.50%	
Lee et al.(2016) (33)	Korea	Cohort Study	HF-WBI	379	49 (26-81)^*b^	I-IIB	HR+ 301 (79.4)	96.80%	IBR, OS, DFS, AE
			CF-WBI	379	50 (27-80)^*b^		HR+ 267 (70.4)	94.40%	
Chuang et al.(2022) (34)	Taiwan,China	Cohort Study	CF-WBI	359	<50 91 ≥50 268	I-IIA	ER+ 284 (79.1) PR+ 259 (72.1)	NR	IBR, OS
			HF-WBI	359	<50 88 ≥50 271		ER+ 292 (81.3) PR+ 254 (70.7)		
Ferraro et al.(2012) (35)	USA	Cohort Study	ISBT	202	60.0 (34.7-84.3)^*b^	0-IA	ER+ 167 (82.7) PR+ 134 (66.3)	100%	IBR, LR, DFS, OS
			CF-WBI	94	56.9 (33.0-83.2)^*b^		ER+ 71 (71.6) PR+ 56 (59.6)	100%	
Wobb et al.(2015) (36)	USA	Cohort Study	Balloon catheter	327	65 (40-91)^*b^	0-IIB	NR	NR	Cosmesis, AE
			CF-WBI	489	61 (32-88)^*b^				
Karasawa et al.(2014) (37)	Japan	Cohort Study	HF-WBI	717	54 (29-85)^*b^	0-IIB	ER+ 557 (78.6) PR+ 483 (65.8)	67.80%	IBR, AE
			CF-WBI	381	53 (22-88)^*b^		ER+ 319 (81.2) PR+ 274 (69.8)	60.30%	
Polgár et al.(2004) (38)	Hungary	Cohort Study	ISBT	45	56 (38-78)^*b^	I-IIA	NR	NR	IBR, OS, cosmesis, AE
			CF-WBI	44	58 (34-78)^*b^				
Deantonio et al.(2016) (39)	Italy	Cohort Study	HF-WBI	85	71.9 (61-86)^*b^	I-IIA	NR	100%	IBR, DFS, OS, cosmesis, AE
			CF-WBI	70	71.6 (61-84)^*b^			100%	
Dong et al.(2021) (40)	China	Cohort Study	HF-WBI	104	<50 80 ≥50 24	0-IIB	HR+ 83 (79.8)	100%	IBR, DFS, OS, cosmesis
			CF-WBI	81	<50 52 ≥50 29		HR+ 60 (74.1)	100%	
Sato et al.(2017) (41)	Japan	Cohort Study	ISBT	99	44 (30-49)^*b^	0-IIB	ER+ 91 (91.9)	86.90%	IBR
			CF-WBI	85	42.4 (31-49)^*b^		ER+ 79 (92.9)	77.60%	
Yoshida-Ichikawa et al.(2021) (42)	Japan	Cohort Study	HF-WBI	186	<50 72 ≥50 114	0-IIB	ER+ 146 (78) PR+ 120 (65)	90%	IBR, OS
			CF-WBI	186	<50 72 ≥50 114		ER+ 154 (83) PR+ 130 (70)	90%	
Lalani et al.(2014) (43)	Canada	Cohort Study	CF-WBI	971	55 (49–64)^*a^	0-IIIA	NR	63%	IBR
			HF-WBI	638	57 (50–66)^*a^			61.10%	
Xu et al.(2025) (44)	China	Cohort Study	IOeRT	21	<50 7 ≥50 14	I-IIA	ER+ 19 (90.5) PR+ 17 (81.0)	100%	IBR, cosmesis
			CF-WBI	38	<50 25 ≥50 13		ER+ 27 (71.1) PR+ 26 (68.4)	100%	

CF-WBI, conventional fractionated whole-breast irradiation; HF-WBI, hypofractionated whole-breast irradiation; UHF-WBI, ultra-hypofractionated whole-breast irradiation; IORT, intraoperative radiotherapy; IOeRT, intraoperative electron radiotherapy; IMRT, intensity-modulated radiation therapy; ISBT, interstitial brachytherapy; 3D-CRT, three-dimensional conformal radiotherapy; IBR, ipsilateral breast recurrence; LR, locoregional recurrence; OS, overall survival; DFS, disease-free survival.

^*a^ Median (IQR).

^*b^ mean(Range).

^*c^ mean ± SD.

### 3.2 Risk of bias

Twenty-three RCTs were assessed using the Cochrane Risk of Bias tool, which includes seven evaluation domains. For random sequence generation, one study was classified as having an unclear risk of bias, whereas the remaining studies were considered as having low risk. Regarding allocation concealment, five studies were rated as having an unclear risk of bias, whereas the others were categorized as low risk.

Because of the substantial heterogeneity among radiotherapy protocols, blinding of participants and personnel was not feasible; therefore, all included studies were considered to have a high risk of bias in this domain. For blinding of outcome assessment, 10 studies were classified as having a high risk, 10 as unclear risk, and 3 as low risk.

**Figure 2:**
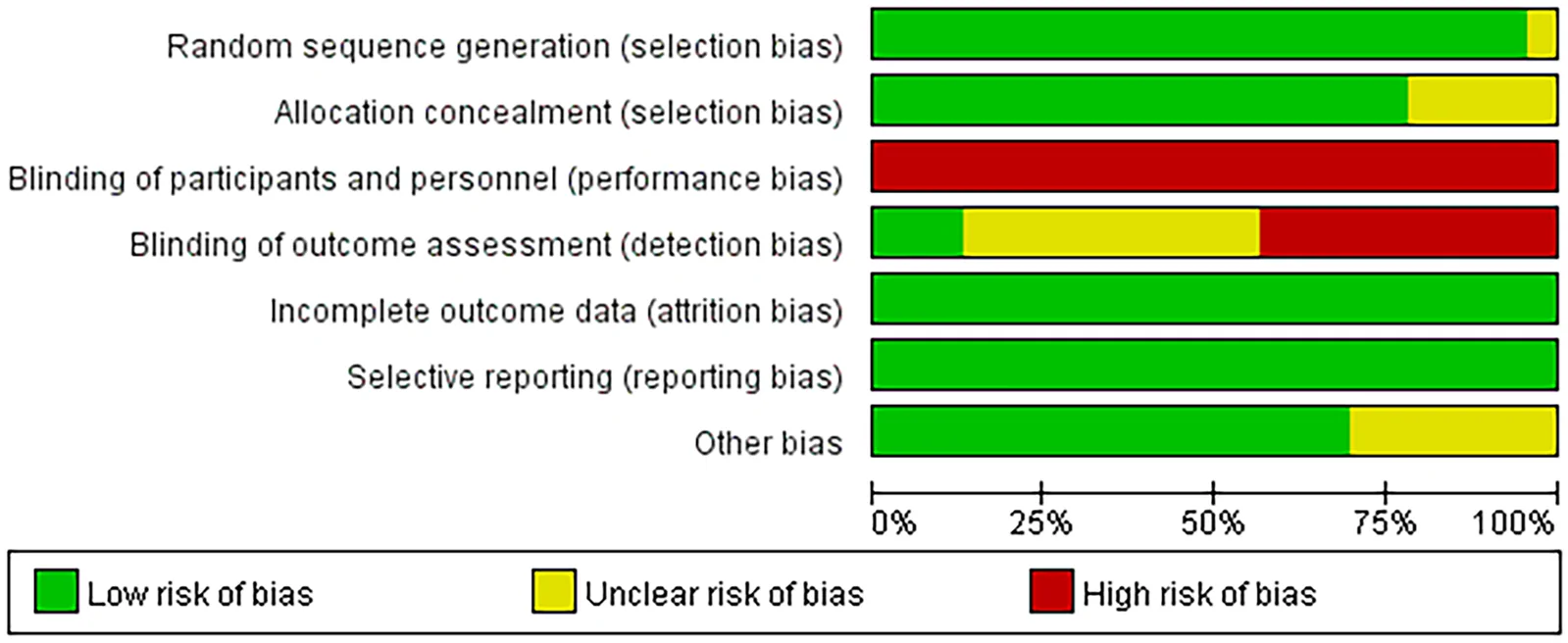
Risks of bias.

All included studies were assessed as having a low risk of bias for incomplete outcome data and selective reporting. Regarding other potential sources of bias, seven studies were categorized as having an unclear risk, whereas the remaining studies were considered low risk. The distribution of risk across all domains is shown in **Figure 2**, and detailed quality assessments for each study are provided in **Supplementary Figure 1**.

Thirteen cohort studies were evaluated using the NOS, which applies a nine-point scoring system. As shown in **Table 2**, five studies achieved the score of 9, four studies scored 8, and the remaining four studies scored 7, indicating an overall high methodological quality among the included cohort studies.

**Table 2:** Methodological quality of prospective case–control studies based on the NOS.

Included studies	Study population selection	Comparability	Exposure or results	Levels
Chi et al. (2024) (32)	☆☆☆☆	☆☆	☆☆	8☆
Lee et al. (2016) (33)	☆☆☆	☆	☆☆☆	7☆
Chuang et al. (2022) (34)	☆☆☆☆	☆☆	☆☆☆	9☆
Ferraro et al. (2012) (35)	☆☆☆☆	☆☆	☆☆☆	9☆
Wobb et al. (2015) (36)	☆☆☆☆	☆	☆☆☆	8☆
Karasawa et al. (2014) (37)	☆☆☆☆	☆	☆☆☆	8☆
Polgár et al. (2004) (38)	☆☆☆☆		☆☆☆	7☆
Deantonio et al. (2016) (39)	☆☆☆☆	☆☆	☆☆☆	9☆
Dong et al. (2021) (40)	☆☆☆☆	☆	☆☆☆	8☆
Sato et al. (2017) (41)	☆☆☆☆		☆☆☆	7☆
Yoshida et al. (2021) (42)	☆☆☆☆	☆☆	☆☆☆	9☆
Lalani et al. (2014) (43)	☆☆☆☆	☆☆	☆☆☆	9☆
Xu et al. (2025) (44)	☆☆☆☆		☆☆☆	7☆

### 3.3 Consistency analysis

Consistency and inconsistency were tested for each outcome, and network consistency was evaluated based on differences in the DIC and effect sizes. The results demonstrated good consistency of the network evidence for IBR, LR, OS, and DFS, indicating that the consistency model was appropriate for these outcomes.

For cosmetic outcomes, the limited number of studies evaluating 3D-CRT and IMRT, together with the presence of extreme outcome values, resulted in poor convergence of the consistency model and abnormal effect estimates in several comparisons. These findings suggested that the consistency model was unreliable for cosmetic outcomes. Therefore, the inconsistency model was adopted for the analysis of cosmetic outcomes (**Supplementary Data 2**).

### 3.4 Outcomes

#### 3.4.1 Ipsilateral breast recurrence

**Figure 3:**
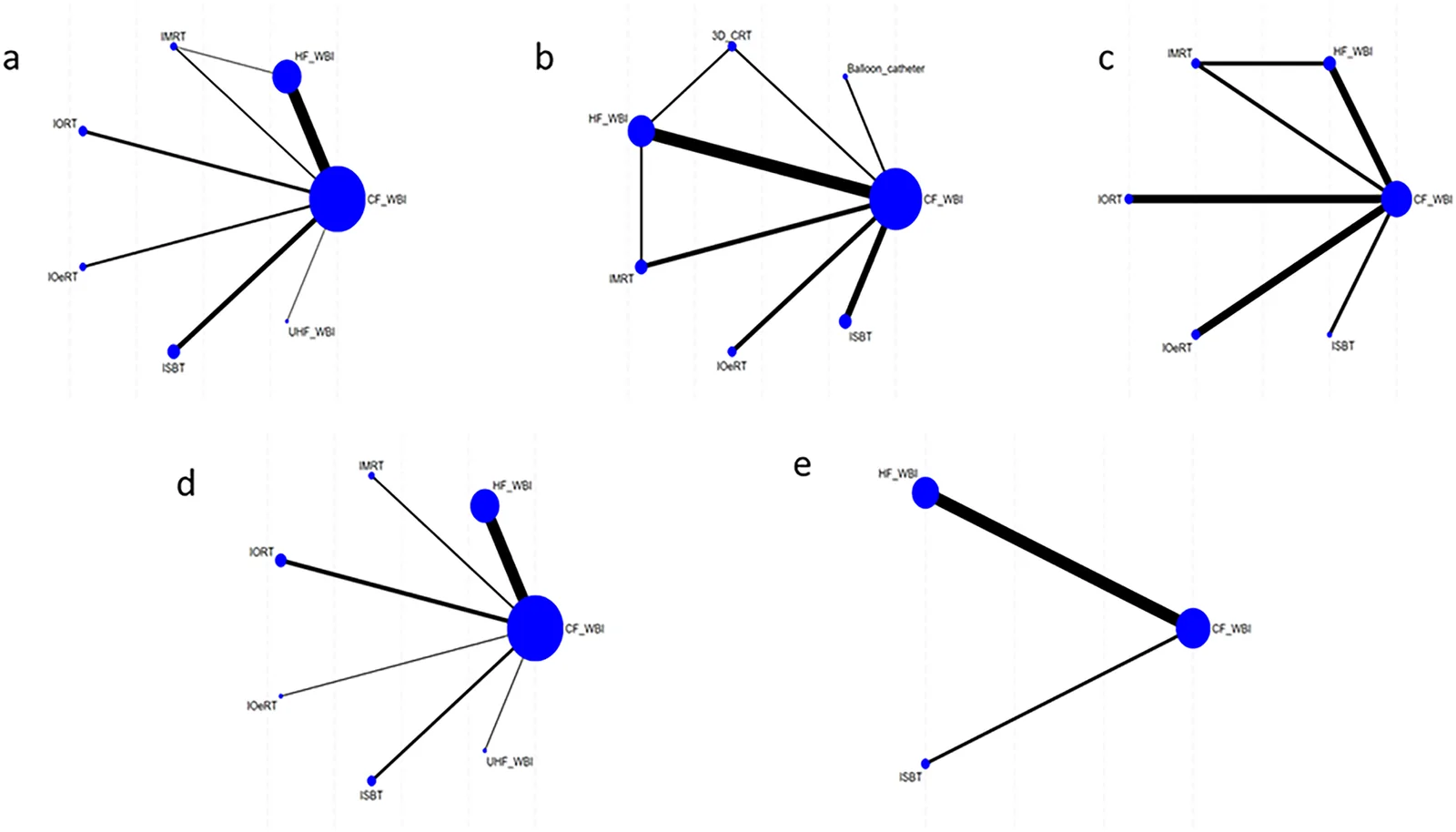
Network of treatment comparisons for **(a)** ipsilateral breast recurrence, **(b)** cosmetic outcomes, **(c)** locoregional recurrence, **(d)** overall survival, and **(e)** disease-free survival. CF-WBI, conventional fractionated whole-breast irradiation; HF-WBI, hypofractionated whole-breast irradiation; UHF-WBI, ultra-hypofractionated whole-breast irradiation; IORT, intraoperative radiotherapy; IOeRT, intraoperative electron radiotherapy; IMRT, intensity-modulated radiation therapy; ISBT, interstitial brachytherapy; 3D-CRT, three-dimensional conformal radiotherapy.

Thirty-one studies involving seven radiotherapy modalities were included in the analysis of IBR. Studies investigating CF-WBI, HF-WBI, and interstitial brachytherapy (ISBT) were the most common. The most frequent comparisons were CF-WBI versus HF-WBI, IORT, and ISBT (**Figure 3a**).

HF-WBI demonstrated the lowest IBR rate (SUCRA = 10.54%), followed by CF-WBI (SUCRA = 35.70%). The remaining interventions were ranked from lower to higher IBR rates as follows: IMRT (SUCRA = 36.64%), ISBT (SUCRA = 45.23%), UHF-WBI (SUCRA = 52.29%), IORT (SUCRA = 72.64%), and IOeRT (SUCRA = 96.96%).

**Figure 4:**
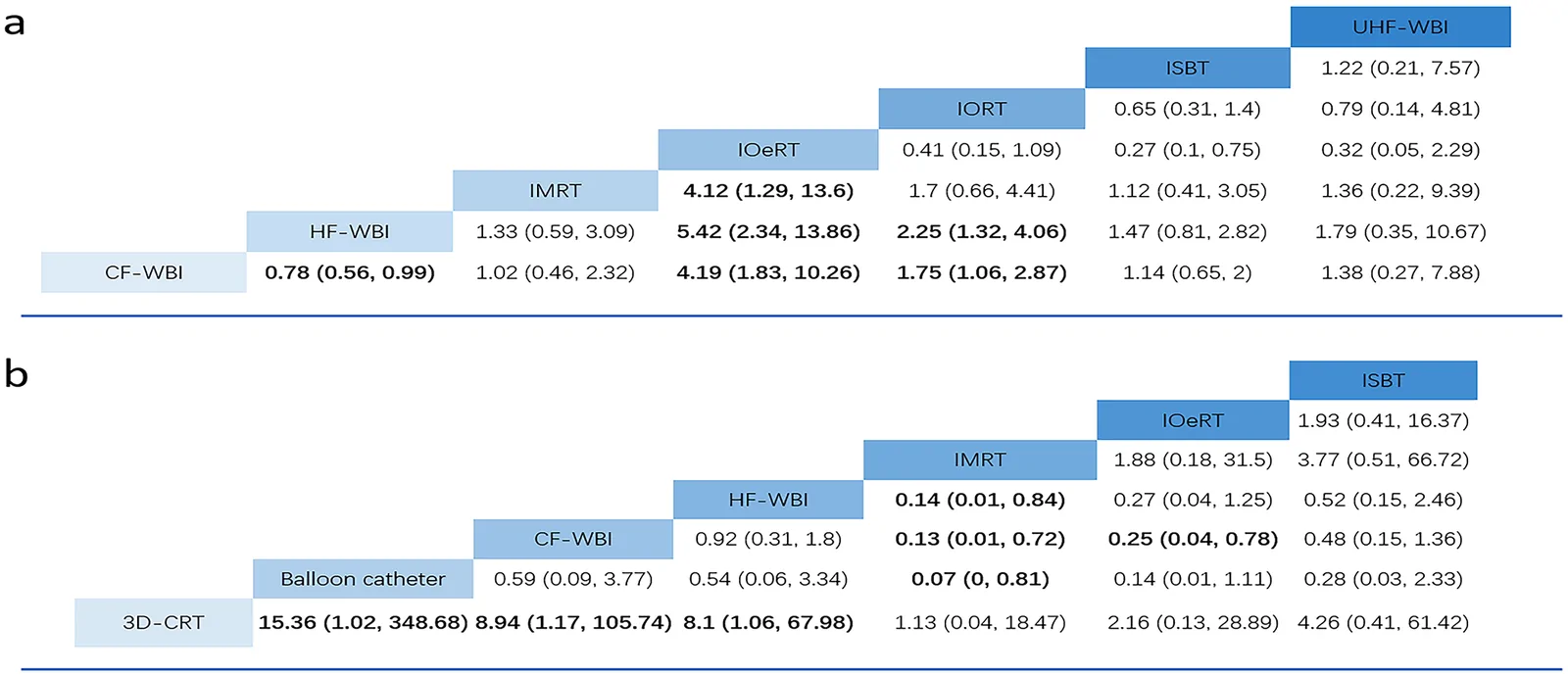
Results of the network meta-analysis for **(a)** ipsilateral breast recurrence and **(b)** cosmesis. CrI, credible interval; CF-WBI, conventional fractionated whole-breast irradiation; HF-WBI, hypofractionated whole-breast irradiation; UHF-WBI, ultra-hypofractionated whole-breast irradiation; IORT, intraoperative radiotherapy; IOeRT, intraoperative electron radiotherapy; IMRT, intensity-modulated radiation therapy; ISBT, interstitial brachytherapy; 3D-CRT, three-dimensional conformal radiotherapy. Each cell represents the comparison (HR and 95% CrI) between the row treatment and the column treatment. All bolded values are statistically significant at p = 0.05.

Compared with CF-WBI, HF-WBI (OR, 0.78; 95% CrI [0.56–0.99]), IOeRT (OR, 4.19; 95% CrI [1.83–10.26]), and IORT (OR, 1.75; 95% CrI [1.06–2.87]) were associated with significantly different IBR rates. Compared with HF-WBI, IOeRT (OR, 5.42; 95% CrI, 2.34–13.86) and IORT (OR, 2.25; 95% CrI, 1.32–4.06) were associated with significantly increased IBR rates. In addition, compared with IMRT, IOeRT was associated with a significantly increased IBR rate (OR, 4.12; 95% CrI, 1.29–13.60) (**Figure 4a**).

#### 3.4.2 Cosmetic outcomes

Sixteen studies met the inclusion criteria for the analysis of cosmetic outcomes, involving seven radiotherapy modalities. Studies investigating CF-WBI, HF-WBI, ISBT, and IMRT were the most common. Furthermore, comparative analyses between CF-WBI and HF-WBI, IMRT, or ISBT represented the most frequent study designs (**Figure 3b**).

The best cosmetic outcomes were demonstrated by 3D-CRT (SUCRA = 14.43%), followed by IMRT (SUCRA = 16.04%). The remaining interventions ranked from lowest to highest for cosmetic outcomes were as follows: IOeRT (SUCRA = 28.52%), ISBT (SUCRA = 48.92%), HF-WBI (SUCRA = 73.25%), CF-WBI (SUCRA = 78.68%), and balloon catheter brachytherapy (SUCRA = 90.17%).

Compared with 3D-CRT, balloon catheter brachytherapy (OR, 15.36; 95% CrI, 1.02–348.68), CF-WBI (OR, 8.94; 95% CrI, 1.17–105.74), and HF-WBI (OR, 8.1; 95% CrI, 1.06–67.98) demonstrated significantly poorer cosmetic outcomes. Compared with balloon catheter brachytherapy, IMRT (OR, 0.07; 95% CrI, 0–0.81) achieved significantly improved cosmetic outcomes. Compared with CF-WBI, IMRT (OR, 0.13; 95% CrI, 0.01–0.72) and IOeRT (OR, 0.25; 95% CrI, 0.04–0.78) demonstrated significantly improved cosmetic outcomes. Furthermore, compared with HF-WBI, IMRT (OR, 0.14; 95% CrI, 0.01–0.84) was associated with significantly improved cosmetic outcomes (**Figure 4b**).

However, the limited number of studies investigating 3D-CRT and IMRT, coupled with the presence of outlier data, resulted in a low level of evidence. Therefore, the reliability of conclusions regarding the effects of these radiotherapy modalities on cosmetic outcomes remains constrained.

#### 3.4.3 LR, OS, and DFS

For the analysis of LR, nine studies evaluating six interventions were included. The evidence network was mainly driven by CF-WBI and HF-WBI, with the most frequent direct comparisons being CF-WBI versus HF-WBI, IORT, or IOeRT. Based on SUCRA values, HF-WBI demonstrated the lowest LR rate (SUCRA = 30.70%), followed closely by CF-WBI (SUCRA = 37.76%). The remaining interventions were ranked from lower to higher LR rates as follows: IMRT (SUCRA = 43.43%), ISBT (SUCRA = 48.23%), IORT (SUCRA = 60.42%), and IOeRT (SUCRA = 79.46%).

For OS, 22 studies involving 7 interventions were included. The network was primarily composed of CF-WBI, HF-WBI, and IORT, with the most common direct comparisons occurring between CF-WBI and either HF-WBI or IORT. IMRT demonstrated the highest probability of reducing mortality risk (SUCRA = 21.94%), followed by IORT (SUCRA = 27.11%). The remaining interventions, ranked according to increasing mortality risk, were ISBT (SUCRA = 45.06%), HF-WBI (SUCRA = 59.89%), CF-WBI (SUCRA = 60.41%), UHF-WBI (SUCRA = 66.91%), and IOeRT (SUCRA = 68.68%).

The DFS network included nine studies comparing three interventions: CF-WBI, HF-WBI, and ISBT. HF-WBI demonstrated the highest probability of achieving the lowest event risk (SUCRA = 35.52%), followed by ISBT (SUCRA = 41.82%) and CF-WBI (SUCRA = 74.65%).

Although the SUCRA rankings suggested a hierarchical trend among treatments, pairwise comparisons revealed no significant differences among interventions for LR, OS, or DFS (**Supplementary Figure 2**).

### 3.5 Adverse events

Due to significant heterogeneity in the criteria for adverse events among the included original studies, this study was unable to conduct a quantitative NMA of AEs. We pooled the extractable data and performed a descriptive analysis (**Supplementary Table 1**).

Total Acute AEs: Lower rates were observed with IMRT (21.1%) and 3D-CRT (32.3%, 21/65), while CF-WBI (66.5%–100.0%) and HF-WBI (43.3%–100.0%) reported higher rates. Grade≥ 2 Acute AEs: Rates were markedly lower in IMRT (2.0%, 5/246) and 3D-CRT (7.7%, 5/65) compared to CF-WBI (37.7% – 77.9%) and HF-WBI (14.9%–47.1%).Acute Skin Toxicity: Lowest in ISBT (13.3%, 6/45) and IMRT (13.4%–19.9%), compared to higher rates in CF-WBI (11.4%–94.8%) and HF-WBI (26.9%–90.1%).

Total Late AEs: Lower rates were observed in HF-WBI (4.7%, 4/85) and IMRT (4.5%, 11/246), compared to higher rates in CF-WBI (5.7%–30.0%).Grade ≥ 2 Late AEs: Rates were markedly lower in IMRT (0.0%, 0/246) and 3D-CRT (0.0%, 0/51), compared to CF-WBI (0.0%–32.4%) and HF-WBI (31.0%, 40/129).Late Skin Toxicity: Lower rates were observed in IOeRT (1.3%, 6/464),ISBT (3.2%–14.3%), and IMRT (4.5%–18.3%) compared to higher rates in CF-WBI (1.2%–29.5%) and HF-WBI (3.3%–41.1%).

Subcutaneous effects ranged from 6.3% to 54.5%, with lower rates in 3D-CRT (6.3%, 4/64) compared to CF-WBI (16.9%–54.5%) and HF-WBI (15.7%–51.9%). Late fibrosis varied widely from 0.0% to 44.4%, with lower rates in CF-WBI (0.2%–36.9%) and HF-WBI (7.4%–41.8%), compared to higher rates in ISBT(0.0%–44.4%). Late pain remained generally low across modalities (≤ 3.7%), with lower rates in Balloon catheter (3.7%, 7/188), compared to specific cohorts of CF-WBI (21.4%,84/393) and ISBT (21.7%,105/484). Late edema ranged from 0.0% to 14.9%, remaining low in IOeRT (0.0%, 0/464) and IMRT (1.6%, 4/246), compared to higher rates in HF-WBI (0%–14.9%). Late hyperpigmentation was lower in Balloon catheter (2.8%, 5/180) compared to HF-WBI (22.4%,15/67). Conversely, late fat necrosis was higher in Interstitial Brachytherapy (9.1%–22.2%) and Balloon catheter (5.8%,19/327), compared to lower rates in CF-WBI (0.0%–17.1%) and IOeRT (1.6%, 7/440).

### 3.6 Funnel plot analysis for publication bias

**Figure 5:**
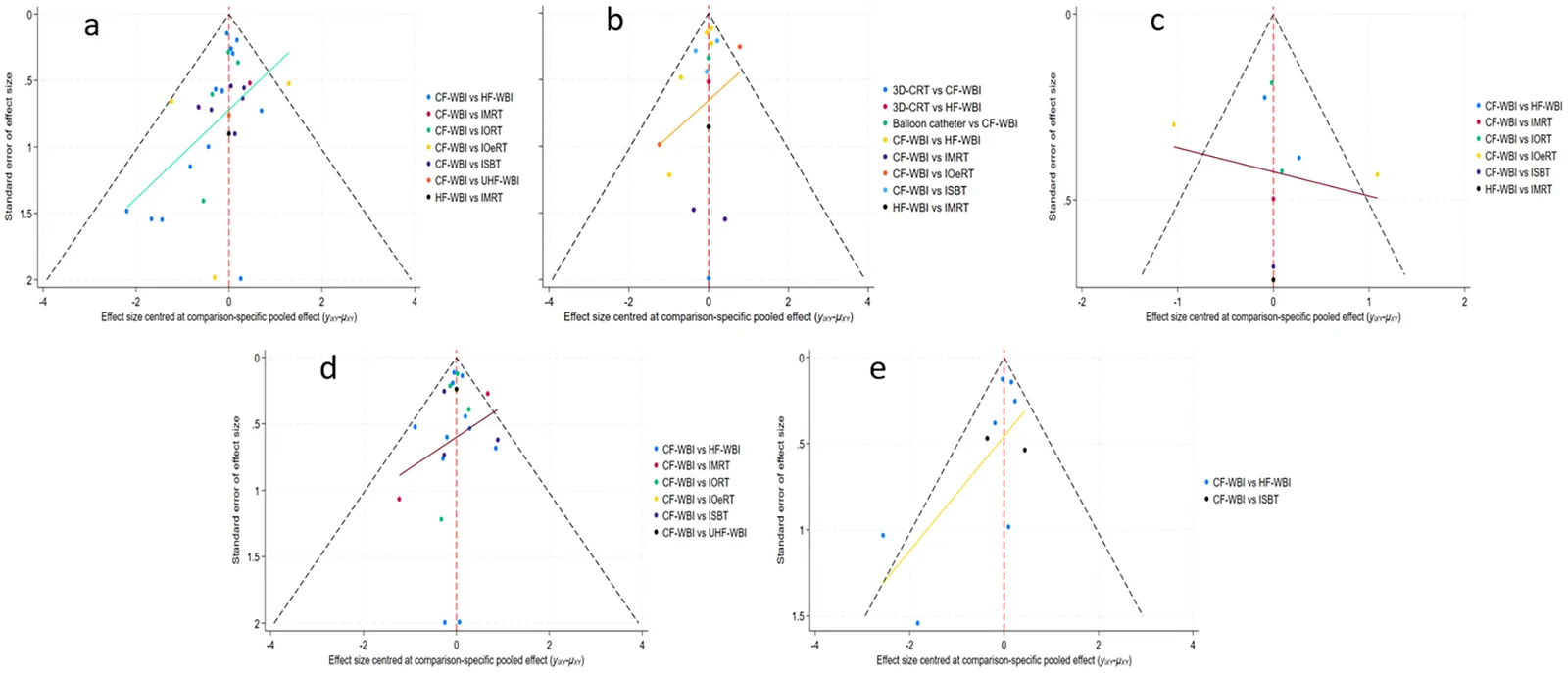
Funnel plots for **(a)** ipsilateral breast recurrence, **(b)** cosmetic outcomes, **(c)** locoregional recurrence, **(d)** overall survival, **(e)** disease-free survival. CF-WBI, conventional fractionation whole-breast irradiation; HF-WBI, hypofractionated whole-breast irradiation; UHF-WBI, ultra-hypofractionated whole-breast irradiation; IORT, intraoperative radiotherapy; IOeRT, intraoperative electron radiotherapy; IMRT, intensity-modulated radiation therapy; ISBT, interstitial brachytherapy; 3D-CRT, three-dimensional conformal radiotherapy.

Funnel plots were generated to evaluate potential publication bias. Overall, the funnel plots for all five outcomes were generally symmetrical, indicating no substantial evidence of publication bias. However, given the limited number of studies available for several specific comparisons, the statistical power of the funnel plot assessment is inherently constrained. Therefore, while no major asymmetry was detected, the presence of mild publication bias or small-study effects cannot be completely ruled out (**Figure 5**).

### 3.7 Network meta-analysis of randomized controlled trials

#### 3.7.1 Ipsilateral breast recurrence in randomized controlled trials

A total of 20 RCTs evaluating seven radiotherapy modalities were included in the RCT-only network (**Supplementary Figure 3a**). Similar to the primary analysis, HF-WBI demonstrated the highest probability of achieving the lowest IBR rate (SUCRA = 13.99%), followed by CF-WBI (SUCRA = 31.80%). The SUCRA hierarchy for the remaining interventions was as follows: IMRT (SUCRA = 35.70%), UHF-WBI (SUCRA = 49.41%), ISBT (SUCRA = 55.68%), IORT (SUCRA = 68.70%), and IOeRT (SUCRA = 95.34%).

Compared with CF-WBI, IOeRT was associated with a significantly increased risk of IBR (OR, 4.15; 95% CrI, 1.49–10.21). When using HF-WBI as the reference, both IOeRT (OR, 5.08; 95% CrI, 1.80–13.74) and IORT (OR, 2.13; 95% CrI, 1.07–4.74) demonstrated significantly higher IBR rates. Furthermore, IOeRT exhibited a significantly higher risk of IBR than IMRT (OR, 3.98; 95% CrI, 1.01–13.53) (**Supplementary Figure 4a**).

#### 3.7.2 Cosmetic outcomes in randomized controlled trials

Eleven RCTs met the inclusion criteria for the analysis of cosmetic outcomes, covering six radiotherapy modalities (**Supplementary Figure 3b**). The highest SUCRA ranking for optimal cosmetic outcomes was retained by 3D-CRT (SUCRA = 13.29%), followed by IMRT (SUCRA = 15.17%). The remaining interventions were ranked as follows: ISBT (SUCRA = 48.04%), IOeRT (SUCRA = 52.25%), CF-WBI (SUCRA = 82.22%), and HF-WBI (SUCRA = 89.02%).

Compared with 3D-CRT, CF-WBI (OR, 7.23; 95% CrI, 1.28–66.92) and HF-WBI (OR, 8.07; 95% CrI, 1.47–53.37) showed significantly reduced cosmetic outcomes. Compared with CF-WBI (OR, 0.15; 95% CrI, 0.01–0.73) and HF-WBI (OR, 0.14; 95% CrI, 0.01–0.7), IMRT demonstrated a significant advantage in terms of cosmetic outcomes (**Supplementary Figure 4b**).

#### 3.7.3 LR, OS, and DFS in randomized controlled trials

The analysis of RCT-only networks included 7 studies evaluating five interventions for LR, 14 studies evaluating seven interventions for OS, and 5 studies evaluating three interventions for DFS. Consistent with the primary pooled analysis, pairwise comparisons showed no significant differences among any evaluated radiotherapy modalities for LR, OS, or DFS (**Supplementary Figure 3c–e**).

The SUCRA ranking results from the NMA based on RCTs showed that HF-WBI had the lowest LR rate (SUCRA = 31.44%), followed by CF-WBI (SUCRA = 38.92%), with the remaining interventions ranked from higher to highest LR rates as follows: IMRT (SUCRA = 44.12%), IORT (SUCRA = 56.33%), and IOeRT (SUCRA = 79.18%). Regarding OS, ISBT exhibited the lowest mortality risk (SUCRA = 25.02%), followed by IMRT (SUCRA = 25.71%). The remaining interventions ranked from lower to higher risk were as follows: IORT (SUCRA = 32.29%), CF-WBI (SUCRA = 63.16%), HF-WBI (SUCRA = 64.29%), UHF-WBI (SUCRA = 68.97%), and IOeRT (SUCRA = 70.55%). For DFS, CF-WBI had the highest probability of achieving a low event risk (SUCRA = 38.62%), followed by ISBT (SUCRA = 54.62%) and HF-WBI (SUCRA = 56.76%).

## 4 Discussion

Radiotherapy currently represents a cornerstone in the management of early-stage BC. By delivering ionizing radiation precisely to breast tissue, radiotherapy effectively eradicates microscopic residual disease, thereby significantly reducing tumor recurrence and cancer-related mortality (3). With continuous advances in medical technology, an increasing number of radiotherapy strategies have been introduced into clinical practice and have demonstrated favorable therapeutic efficacy. In this study, an NMA was conducted to compare the efficacy and cosmetic outcomes of different radiotherapy strategies for early-stage BC. By evaluating multiple clinical outcomes, this study aimed to identify the most effective radiotherapy approaches for patients with early-stage BC.

The results for IBR demonstrated that compared with CF-WBI, HF-WBI was associated with a lower IBR rate, consistent with previous studies. In contrast, patients treated with IORT or IOeRT exhibited significantly higher IBR rates than those receiving CF-WBI or HF-WBI, which is also consistent with earlier reports (29, 45, 46). Furthermore, other radiotherapy modalities, including IMRT, ISBT, and UHF-WBI, did not demonstrate statistically significant differences compared with CF-WBI. Overall, these findings indicate that HF-WBI is the optimal strategy for reducing IBR in patients with early-stage BC.

HF-WBI generally delivers larger radiation doses per fraction over a shorter treatment period, thereby improving patient convenience and optimizing healthcare resource utilization. Previous clinical evidence has established HF-WBI as a standard treatment option for early-stage BC. This approach not only reduces tumor recurrence and metastatic progression but also decreases cumulative radiation exposure to adjacent normal tissues, thereby minimizing treatment-associated toxicities and demonstrating a favorable safety profile (47–49).

Regarding cosmetic outcomes following postoperative radiotherapy, 3D-CRT, IMRT, and IOeRT demonstrated significantly better cosmetic effects than CF-WBI, with 3D-CRT showing the most favorable results. These findings suggest that APBI techniques may provide improved dose homogeneity within the target region, thereby reducing radiation-induced alterations in breast appearance, which is consistent with previous studies (50–52). Three-dimensional conformal radiotherapy is an important radiotherapy modality that uses computed tomography or magnetic resonance imaging to reconstruct accurate 3D contours of the tumor and adjacent normal tissues. By using multileaf collimator technology, radiation beams can be precisely shaped to conform to tumor geometry, thereby maximizing radiation delivery to the target while minimizing unnecessary exposure to adjacent healthy tissues. Consequently, 3D-CRT has become a widely adopted radiotherapy technique in modern oncological practice (53, 54). APBI techniques represented by 3D-CRT can improve target dose uniformity and effectively reduce radiation exposure to normal tissues, thereby minimizing changes in breast appearance after treatment (55, 56).

Based on these findings, IMRT and 3D-CRT may be preferrable radiotherapy strategies when preservation of breast cosmetic outcomes is a primary clinical objective. However, the limited number of studies evaluating 3D-CRT and IMRT, together with the presence of outlier data, resulted in wide credible intervals and low evidence strength in the inconsistency model analysis. Therefore, the effects of different radiotherapy modalities on cosmetic outcomes should be interpreted cautiously, and additional direct comparative studies are needed to validate these findings.

Our analysis revealed no significant differences in local recurrence, OS, or DFS among the evaluated radiotherapy regimens. In the descriptive analysis of AEs, the overall incidence of adverse events was lower with APBI than with WBI; this difference is most likely attributable to improved target contouring and reduced unnecessary radiation exposure to surrounding healthy skin and subcutaneous tissue. These findings support the safety and efficacy of modern radiotherapy approaches, indicating that strategies designed to shorten treatment duration, such as HF-WBI, or reduce the irradiated target volume, such as APBI, provide long-term survival outcomes comparable to those of conventional fractionated whole-breast radiotherapy in patients with early-stage BC. These findings are consistent with the long-term outcomes reported in recent large-scale RCTs (45). Considering that APBI and low-fractionation radiotherapy may improve cosmetic outcomes and reduce the incidence of adverse reactions without affecting survival rates, these approaches warrant greater consideration during the radiotherapy planning process. Nevertheless, the absence of significant differences should be interpreted cautiously. Early-stage BC is generally associated with favorable prognostic outcomes, including low mortality and recurrence rates. Therefore, the available sample sizes and follow-up durations may have provided inadequate statistical power to detect modest differences between interventions when event rates are low.

To assess the potential impact of study design and minimize the selection bias inherent in observational data, we conducted an analysis that included only RCTs. Encouragingly, the results of this pure RCT analysis showed a high degree of consistency with our main NMA across all clinical endpoints. Specifically, HF-WBI and CF-WBI continued to demonstrate superior efficacy in reducing IBR. Regarding OS and LR, the pure RCT analysis consistently showed no statistically significant differences among the various radiotherapy regimens, reaffirming the long-term oncological safety of APBI techniques; however, the SUCRA rankings for DFS differed significantly from those in the main analysis, likely due to the limited number of relevant studies. Furthermore, regarding cosmetic outcomes, APBI based on IMRT and 3D-CRT still retained a statistical advantage; however, due to the limited number of RCTs reporting standardized cosmetic outcomes, the credible intervals remain wide. Overall, the high consistency between the main analysis model and the pure RCT dataset highlights the statistical robustness of our study’s conclusions, confirming that the inclusion of high-quality observational studies did not introduce significant bias into the primary clinical conclusions.

To the best of our knowledge, this study is the first NMA to comprehensively compare CF-WBI, HF-WBI, UHF-WBI, IMRT, ISBT, IORT, IOeRT, 3D-CRT, and balloon catheter radiotherapy, thereby evaluating both their direct and indirect associations.

Nevertheless, this study has several limitations. First, certain networks exhibit a star-shaped configuration dominated by CF-WBI without closed loops, which limits the rigorous evaluation of local consistency and transitivity across indirect comparisons. Second, the limited number of studies for several interventions and the presence of outlier data reduced the precision and stability of the cosmetic outcome analyses. Furthermore, while both WBI and APBI can utilize 3D-CRT and IMRT techniques, this study included only APBI treatments based on 3D-CRT and IMRT, which limits the comprehensiveness of this study.Finally, because of limited available data, some clinically relevant outcomes, including radiotherapy-related adverse events, could not be evaluated, therefore, only descriptive analyses were performed.

## 5 Conclusion

Each of the nine therapeutic strategies evaluated in this study demonstrated distinct advantages in the management of early-stage BC. Based on the findings of the NMA and SUCRA rankings, HF-WBI and CF-WBI appeared to be the most favorable approaches for reducing IBR. In terms of cosmetic outcomes, 3D-CRT and IMRT demonstrated superior performance; However, given the scarcity of relevant data and the wide credible intervals, any conclusions regarding cosmetic effects must be interpreted with caution. For LR, OS, and DFS, the efficacy of all evaluated interventions was comparable.

Therefore, radiotherapy strategies should be individualized according to patient characteristics and specific clinical requirements. Further large-scale, multicenter, high-quality RCTs are necessary to validate these findings and strengthen the evidence supporting different radiotherapy approaches for early-stage BC.

## Data Availability

The original contributions presented in the study are included in the article/**Supplementary Material**. Further inquiries can be directed to the corresponding author.
