# Supplementary material for: Efficacy and cosmetic outcomes of radiotherapy modalities in early-stage breast cancer: a systematic review and network meta-analysis
Source: Front Oncol. 2026 Sep 9;16:1876930. doi: 10.3389/fonc.2026.1876930 (PMC13597323; doi:10.3389/fonc.2026.1876930)
Supplement: Supplementary file 1 [file DataSheet1.pdf]

Supplementary Material

Efficacy and Safety of Acupuncture-Point Stimulation Combined with Opioids for Treatment of Moderate to Severe Cancer Pain: A Network Meta-Analysis of Randomized Controlled Trials

Yushan Dong1, Tingting Liu1, Peng Wang2, Songjiang Liu3*

Authors' affiliations:

[1] Heilongjiang University of Chinese Medicine, Harbin, China

[1] Heilongjiang University of Chinese Medicine, Harbin, China

[2] The First Affiliated Hospital of Heilongjiang University of Chinese Medicine, Harbin, China

[3] The First Affiliated Hospital of Heilongjiang University of Chinese Medicine, Harbin, China*

*** Correspondence:**
Songjiang Liu
liusongjiang@hljucm.edu.cn

1 Supplementary Data

Data 1 The search strategy for each database

PubMed search strategy

Search Date: November 18, 2025

--

#1: (Breast Neoplasms[MeSH Terms]) OR (reast Neoplasm[Title/Abstract] OR Breast Tumor*[Title/Abstract] OR Breast Cancer[Title/Abstract] OR Breast Malignant Neoplasm*[Title/Abstract] OR Breast Malignant Tumor*[Title/Abstract] OR Mammary Cancer*[Title/Abstract] OR Breast Carcinoma*[Title/Abstract])

#2: (Whole-breast irradiation[MeSH Terms]) OR (whole breast irradiat*[Title/Abstract] OR whole-breast radiotherapy*[Title/Abstract] OR WBI[Title/Abstract]) 2,981

#3: (Accelerated Partial Breast Irradiation[MeSH Terms]) OR (Accelerated Partial Breast Irradiations[Title/Abstract] OR APBI[Title/Abstract] OR Interstitial Brachytherapy*[Title/Abstract] OR Balloon-based Brachytherapy*[Title/Abstract] OR IORT[Title/Abstract] OR Electron IORT[Title/Abstract] OR IMRT[Title/Abstract] OR 3D-CRT[Title/Abstract])

#4: #2 OR #3

#5: #1 AND #4

EMBASE search strategy

Search Date: November 18, 2025

--

#1: 'breast tumor'/ex

#2: 'whole breast irradiation'/exp

#3: 'accelerated partial breast irradiation'/exp

#4: 'breast neoplasm':ab,ti OR 'breast tumor*':ab,ti OR 'breast cancer':ab,ti OR 'breast malignant neoplasm*':ab,ti OR 'breast malignant tumor*':ab,ti OR 'mammary cancer*':ab,ti OR 'breast carcinoma*':ab,ti

#5: 'whole breast irradiat*':ab,ti OR 'whole-breast radiotherapy*':ab,ti OR wbi:ab,ti

#6: 'accelerated partial breast irradiations':ab,ti OR apbi:ab,ti OR 'interstitial brachytherapy*':ab,ti OR 'balloon-based brachytherapy*':ab,ti OR iort:ab,ti OR 'electron iort':ab,ti OR imrt:ab,ti OR '3d crt':ab,ti

#7:#1 OR #4

#8:#2 OR #3 OR #5 OR #6

#9:#7 AND #8

Web of Science

Search Date: November 18, 2025

(TS=(Breast Neoplasms OR Breast Neoplasm OR Breast Tumor* OR Breast Cancer OR Breast Malignant Neoplasm* OR Breast Malignant Tumor* OR Mammary Cancer* OR Breast Carcinoma*)) AND TS=(Whole-breast irradiation OR whole breast irradiat* OR whole-breast radiotherapy* OR WBI OR Accelerated Partial Breast Irradiation OR Accelerated Partial Breast Irradiations OR APBI OR Interstitial Brachytherapy* OR Balloon-based Brachytherapy* OR IORT OR Electron IORT OR IMRT OR 3D-CRT)

The Cochrane Library

Search Date: November 18, 2025

#1: MeSH descriptor: [Breast Neoplasms] explode all trees

#2: (Breast Neoplasm OR Breast Tumor* OR Breast Cancer OR Breast Malignant Neoplasm* OR Breast Malignant Tumor* OR Mammary Cancer* OR Breast Carcinoma*):ti,ab,kw

#3: (Whole-breast irradiation OR whole breast irradiat* OR whole-breast radiotherapy* OR WBI):ti,ab,kw

#4：(Accelerated Partial Breast Irradiation OR Accelerated Partial Breast Irradiations OR APBI OR Interstitial Brachytherapy* OR Balloon-based Brachytherapy* OR IORT OR Electron IORT OR IMRT OR 3D-CRT):ti,ab,kw

#5：#1 OR #2

#6：#3 OR #4

#7：#5 AND #6

--

Data 2 Evaluation of consistency and inconsistency

RCT

IBR-RCT

Consistency

Results on the Log Odds Ratio scale

Iterations = 20001:70000

Thinning interval = 1

Number of chains = 4

Sample size per chain = 50000

1. Empirical mean and standard deviation for each variable,

plus standard error of the mean:

	Mean	SD	Naive SE	Time-series SE
d.CF_WBI.HF_WBI	-0.21594	0.2309	0.0005164	0.002592
d.CF_WBI.IMRT	0.04076	0.4514	0.0010093	0.006788
d.CF_WBI.IOeRT	1.40218	0.4798	0.0010728	0.006730
d.CF_WBI.IORT	0.55284	0.2974	0.0006651	0.003003
d.CF_WBI.ISBT	0.36245	0.4067	0.0009094	0.005622
d.CF_WBI.UHF_WBI	0.30810	0.8928	0.0019963	0.014935
sd.d	0.27260	0.2266	0.0005066	0.005333

2. Quantiles for each variable:

	2.5%	25%	50%	75%	97.5%

d.CF_WBI.HF_WBI -0.748456 -0.33702 -0.19401 -0.0704 0.1801

d.CF_WBI.IMRT -0.841650 -0.25453 0.03701 0.3321 0.9394

d.CF_WBI.IOeRT 0.398635 1.10835 1.42224 1.7096 2.3235

d.CF_WBI.IORT -0.057864 0.37736 0.55698 0.7353 1.1295

d.CF_WBI.ISBT -0.416253 0.09507 0.35419 0.6248 1.1889

d.CF_WBI.UHF_WBI -1.456374 -0.26848 0.30316 0.8707 2.1049

sd.d 0.007945 0.09798 0.21745 0.3915 0.8375

-- Model fit (residual deviance):

Dbar pD DIC

39.79690 28.81935 68.61625

40 data points, ratio 0.9949, I^2 = 2%

Inconsistency

Results on the Log Odds Ratio scale

Iterations = 20001:70000

Thinning interval = 1

Number of chains = 4

Sample size per chain = 50000

1. Empirical mean and standard deviation for each variable,

plus standard error of the mean:

	Mean	SD	Naive SE	Time-series SE
d.CF_WBI.HF_WBI	-0.21170	0.2486	0.0005559	0.002622
d.CF_WBI.IMRT	-0.02126	0.5117	0.0011441	0.006381
d.CF_WBI.IOeRT	1.37768	0.4952	0.0011072	0.007122
d.CF_WBI.IORT	0.53915	0.3124	0.0006985	0.003124
d.CF_WBI.ISBT	0.36294	0.4114	0.0009200	0.004996
d.CF_WBI.UHF_WBI	0.33122	0.9129	0.0020413	0.014279
d.HF_WBI.IMRT	0.63541	1.1260	0.0025177	0.022662

sd.d 0.29638 0.2468 0.0005518 0.005573

2. Quantiles for each variable:

	2.5%	25%	50%	75%	97.5%
d.CF_WBI.HF_WBI	-0.784435	-0.33793	-0.18999	-0.0577	0.2190
d.CF_WBI.IMRT	-1.059412	-0.34640	-0.01254	0.3131	0.9593
d.CF_WBI.IOeRT	0.351521	1.07581	1.39025	1.6967	2.3204
d.CF_WBI.IORT	-0.092964	0.35091	0.54254	0.7320	1.1409
d.CF_WBI.ISBT	-0.435014	0.09803	0.35488	0.6282	1.1752
d.CF_WBI.UHF_WBI	-1.422245	-0.26878	0.31238	0.9117	2.2096
d.HF_WBI.IMRT	-1.462485	-0.11171	0.59997	1.3379	3.0231
sd.d	0.008725	0.10791	0.23801	0.4216	0.9139

-- Model fit (residual deviance):

Dbar pD DIC

40.69580 30.21284 70.90864

40 data points, ratio 1.017, I^2 = 4%

LR-RCT

Consistency

Results on the Log Odds Ratio scale

Iterations = 20001:70000

Thinning interval = 1

Number of chains = 4

Sample size per chain = 50000

1. Empirical mean and standard deviation for each variable,

plus standard error of the mean:

Mean SD Naive SE Time-series SE

d.CF_WBI.HF_WBI	-0.19126	0.8039	0.0017976	0.002578
d.CF_WBI.IMRT	0.06281	1.0350	0.0023144	0.003815
d.CF_WBI.IOeRT	0.95850	0.8983	0.0020088	0.003233
d.CF_WBI.IORT	0.38835	1.2183	0.0027241	0.002894
sd.d	1.13331	0.4137	0.0009251	0.002626

2. Quantiles for each variable:

	2.5%	25%	50%	75%	97.5%
d.CF_WBI.HF_WBI	-1.8515	-0.6616	-0.18598	0.2857	1.438
d.CF_WBI.IMRT	-2.0309	-0.5665	0.05825	0.6883	2.170
d.CF_WBI.IOeRT	-0.8599	0.4242	0.95133	1.4895	2.819
d.CF_WBI.IORT	-2.1163	-0.3149	0.38622	1.0930	2.909
sd.d	0.4304	0.8082	1.09971	1.4479	1.920

-- Model fit (residual deviance):

Dbar pD DIC

14.18341 13.75565 27.93906

14 data points, ratio 1.013, I^2 = 8%

Inconsistency

Results on the Log Odds Ratio scale

Iterations = 20001:70000

Thinning interval = 1

Number of chains = 4

Sample size per chain = 50000

1. Empirical mean and standard deviation for each variable,

plus standard error of the mean:

Mean SD Naive SE Time-series SE

d.CF_WBI.HF_WBI	-0.0431	0.9403	0.0021027	0.002439
d.CF_WBI.IMRT	-0.2729	1.3951	0.0031195	0.004235
d.CF_WBI.IOeRT	0.9558	0.9516	0.0021278	0.003228
d.CF_WBI.IORT	0.3888	1.3012	0.0029097	0.003129
d.HF_WBI.IMRT	0.7057	1.5153	0.0033882	0.006686
sd.d	1.2214	0.4165	0.0009312	0.002596

2. Quantiles for each variable:

	2.5%	25%	50%	75%	97.5%
d.CF_WBI.HF_WBI	-1.9719	-0.6006	-0.04646	0.5153	1.886
d.CF_WBI.IMRT	-3.1044	-1.1250	-0.27354	0.5754	2.564
d.CF_WBI.IOeRT	-0.9773	0.3841	0.95073	1.5236	2.915
d.CF_WBI.IORT	-2.2895	-0.3712	0.38442	1.1500	3.071
d.HF_WBI.IMRT	-2.3117	-0.2455	0.69372	1.6497	3.773
sd.d	0.4706	0.8948	1.21294	1.5600	1.941

-- Model fit (residual deviance):

Dbar pD DIC

14.33582 14.06135 28.39717

14 data points, ratio 1.024, I^2 = 9%

<u>OS-RCT</u>

Consistency

Results on the Log Odds Ratio scale

Iterations = 20001:70000

Thinning interval = 1

Number of chains = 4

Sample size per chain = 50000

1. Empirical mean and standard deviation for each variable,

plus standard error of the mean:

	Mean	SD	Naive SE	Time-series SE
d.CF_WBI.HF_WBI	0.01136	0.1467	0.0003279	0.001287
d.CF_WBI.IMRT	-0.33606	0.3547	0.0007932	0.005351
d.CF_WBI.IOeRT	0.10232	0.3463	0.0007744	0.003644
d.CF_WBI.IORT	-0.19548	0.1856	0.0004149	0.001469
d.CF_WBI.ISBT	-0.33146	0.3594	0.0008037	0.003984
d.CF_WBI.UHF_WBI	0.09438	0.3593	0.0008034	0.003898
sd.d	0.16153	0.1670	0.0003733	0.004192

2. Quantiles for each variable:

	2.5%	25%	50%	75%	97.5%
d.CF_WBI.HF_WBI	-0.275004	-0.06884	0.01159	0.09040	0.2947
d.CF_WBI.IMRT	-1.064716	-0.54517	-0.32477	-0.10493	0.3078
d.CF_WBI.IOeRT	-0.571594	-0.10233	0.10490	0.30935	0.7677

d.CF_WBI.IORT   -0.543007 -0.29851 -0.19896 -0.09552 0.1731

d.CF_WBI.ISBT   -1.013962 -0.54856 -0.33215 -0.11544 0.3646

d.CF_WBI.UHF_WBI -0.586099 -0.12330 0.09424 0.30805 0.7909

sd.d   0.005408 0.05252 0.11550 0.21373 0.5988

-- Model fit (residual deviance):

Dbar pD DIC

25.77133 20.98147 46.75280

28 data points, ratio 0.9204, I^2 = 0%

Inconsistency

Results on the Log Odds Ratio scale

Iterations = 20001:70000

Thinning interval = 1

Number of chains = 4

Sample size per chain = 50000

1. Empirical mean and standard deviation for each variable,

plus standard error of the mean:

```
                  Mean     SD  Naive SE Time-series SE
d.CF_WBI.HF_WBI   0.01011 0.1407 0.0003145       0.001272
d.CF_WBI.IMRT    -0.32397 0.3453 0.0007720       0.005386
d.CF_WBI.IOeRT    0.10489 0.3352 0.0007496       0.003622
d.CF_WBI.IORT    -0.19347 0.1778 0.0003977       0.001485
d.CF_WBI.ISBT    -0.33293 0.3480 0.0007782       0.003876
d.CF_WBI.UHF_WBI  0.10379 0.3461 0.0007738       0.003939
sd.d              0.15466 0.1491 0.0003335       0.003097
```

2. Quantiles for each variable:

	2.5%	25%	50%	75%	97.5%
d.CF_WBI.HF_WBI	-0.264360	-0.06994	0.009528	0.09060	0.2852
d.CF_WBI.IMRT	-1.028767	-0.53034	-0.310413	-0.09589	0.3147
d.CF_WBI.IOeRT	-0.544412	-0.09976	0.102320	0.30948	0.7642
d.CF_WBI.IORT	-0.534470	-0.29521	-0.197017	-0.09576	0.1724
d.CF_WBI.ISBT	-1.012833	-0.54734	-0.331621	-0.11299	0.3354
d.CF_WBI.UHF_WBI	-0.565661	-0.11117	0.104881	0.31791	0.7738
sd.d	0.004808	0.05186	0.114241	0.20961	0.5503

-- Model fit (residual deviance):

Dbar	pD	DIC
25.76261	20.91427	46.67688

28 data points, ratio 0.9201, I^2 = 0%

DFs-RCT

Consistency：

Results on the Log Odds Ratio scale

Iterations = 20001:70000

Thinning interval = 1

Number of chains = 4

Sample size per chain = 50000

1. Empirical mean and standard deviation for each variable,

plus standard error of the mean:

	Mean	SD	Naive SE	Time-series SE
d.CF_WBI.HF_WBI	0.05006	0.12162	0.0002719	0.0011958
d.CF_WBI.ISBT	0.08383	0.55859	0.0012490	0.0116572
sd.d	0.10115	0.06085	0.0001361	0.0006631

2. Quantiles for each variable:

	2.5%	25%	50%	75%	97.5%
d.CF_WBI.HF_WBI	-0.187328	-0.02981	0.04851	0.1293	0.2948
d.CF_WBI.ISBT	-1.030918	-0.28513	0.09033	0.4491	1.1588
sd.d	0.005011	0.04865	0.09808	0.1516	0.2089

-- Model fit (residual deviance):

Dbar	pD	DIC
8.296452	7.436612	15.733064

10 data points, ratio 0.8296, I^2 = 0%

Inconsistency：

Results on the Log Odds Ratio scale

Iterations = 20001:70000

Thinning interval = 1

Number of chains = 4

Sample size per chain = 50000

1. Empirical mean and standard deviation for each variable,

plus standard error of the mean:

	Mean	SD	Naive SE	Time-series SE
d.CF_WBI.HF_WBI	0.05124	0.12025	0.0002689	0.0011626
d.CF_WBI.ISBT	0.08690	0.56783	0.0012697	0.0118866
sd.d	0.09974	0.06059	0.0001355	0.0006571

2. Quantiles for each variable:

	2.5%	25%	50%	75%	97.5%
d.CF_WBI.HF_WBI	-0.181186	-0.02831	0.04985	0.1288	0.2941
d.CF_WBI.ISBT	-1.029072	-0.28701	0.07731	0.4616	1.2211

sd.d 0.004959 0.04754 0.09593 0.1497 0.2084

-- Model fit (residual deviance):

Dbar	pD	DIC
8.281200	7.409299	15.690499

10 data points, ratio 0.8281, I^2 = 0%

<u>Cosmesis-RCT</u>

Consistency：

Results on the Log Odds Ratio scale

Iterations = 20001:70000

Thinning interval = 1

Number of chains = 4

Sample size per chain = 50000

1. Empirical mean and standard deviation for each variable,

plus standard error of the mean:

	Mean	SD	Naive SE	Time-series SE
d.CF_WBI.3D_CRT	-2.03759	0.9911	0.0022162	0.011761
d.CF_WBI.HF_WBI	0.07638	0.4130	0.0009234	0.003240
d.CF_WBI.IMRT	-1.99542	1.0375	0.0023200	0.016716
d.CF_WBI.IOeRT	-0.62562	0.7445	0.0016648	0.003181
d.CF_WBI.ISBT	-0.71178	0.5162	0.0011542	0.002127
sd.d	0.49714	0.4512	0.0010088	0.008257

2. Quantiles for each variable:

	2.5%	25%	50%	75%	97.5%
d.CF_WBI.3D_CRT	-4.20355	-2.55439	-1.9778	-1.4528	-0.2463
d.CF_WBI.HF_WBI	-0.94625	-0.06613	0.1239	0.2833	0.7636

d.CF_WBI.IMRT   -4.37708 -2.56021 -1.8862 -1.2919 -0.3121

d.CF_WBI.IOeRT -2.15854 -0.97531 -0.6234 -0.2756 0.9000

d.CF_WBI.ISBT   -1.84882 -0.93208 -0.6883 -0.4691 0.2953

sd.d   0.01966 0.17886 0.3615 0.6642 1.7926

-- Model fit (residual deviance):

    Dbar   pD   DIC

21.56988 17.59559 39.16547

22 data points, ratio 0.9804, I^2 = 3%

Inconsistency：

Results on the Log Odds Ratio scale

Iterations = 20001:70000

Thinning interval = 1

Number of chains = 4

Sample size per chain = 50000

1. Empirical mean and standard deviation for each variable,

plus standard error of the mean:

	Mean	SD	Naive SE	Time-series SE
d.3D_CRT.CF_WBI	-19.0412	24.4836	0.0547471	4.052510
d.3D_CRT.HF_WBI	2.1111	0.8456	0.0018908	0.008655
d.CF_WBI.HF_WBI	0.1889	0.3625	0.0008105	0.001334
d.CF_WBI.IMRT	-28.9160	18.5009	0.0413692	3.525098
d.CF_WBI.IOeRT	-0.6186	0.6696	0.0014972	0.003226
d.CF_WBI.ISBT	-0.7093	0.4641	0.0010379	0.002149
d.HF_WBI.IMRT	-0.7655	1.1349	0.0025377	0.014049
sd.d	0.4313	0.3974	0.0008887	0.006721

2. Quantiles for each variable:

	2.5%	25%	50%	75%	97.5%
d.3D_CRT.CF_WBI	-69.62616	-36.92426	-16.5167	-0.66127	23.6753
d.3D_CRT.HF_WBI	0.51890	1.60616	2.0751	2.59702	3.8545
d.CF_WBI.HF_WBI	-0.55120	0.03023	0.1874	0.34573	0.9382
d.CF_WBI.IMRT	-67.50442	-42.68265	-24.9389	-13.87221	-3.3599
d.CF_WBI.IOeRT	-1.96659	-0.94383	-0.6203	-0.28933	0.7326
d.CF_WBI.ISBT	-1.70272	-0.91685	-0.6902	-0.47941	0.1782
d.HF_WBI.IMRT	-3.09882	-1.45145	-0.7337	-0.03985	1.3896
sd.d	0.01451	0.15486	0.3172	0.57571	1.5756

-- Model fit (residual deviance):

Dbar	pD	DIC
18.50490	17.34961	35.85451

22 data points, ratio 0.8411, I^2 = 0%

Joint Aanlysis

IBR

Consistency：

Results on the Log Odds Ratio scale

Iterations = 20001:70000

Thinning interval = 1

Number of chains = 4

Sample size per chain = 50000

1. Empirical mean and standard deviation for each variable,

plus standard error of the mean:

Mean SD Naive SE Time-series SE

d.CF_WBI.HF_WBI	-0.25935	0.1433	0.0003204	0.002203
d.CF_WBI.IMRT	0.02388	0.4169	0.0009322	0.008259
d.CF_WBI.IOeRT	1.44852	0.4374	0.0009781	0.009522
d.CF_WBI.IORT	0.55928	0.2522	0.0005640	0.003491
d.CF_WBI.ISBT	0.13494	0.2881	0.0006443	0.005696
d.CF_WBI.UHF_WBI	0.34420	0.8676	0.0019400	0.020795
sd.d	0.17699	0.1479	0.0003307	0.004448

2. Quantiles for each variable:

	2.5%	25%	50%	75%	97.5%
d.CF_WBI.HF_WBI	-0.574575	-0.34162	-0.25019	-0.1655	-0.005183
d.CF_WBI.IMRT	-0.772966	-0.26148	0.02386	0.3076	0.839606
d.CF_WBI.IOeRT	0.606515	1.16001	1.43363	1.7364	2.327992
d.CF_WBI.IORT	0.056820	0.40235	0.56032	0.7172	1.055190
d.CF_WBI.ISBT	-0.424726	-0.05926	0.13278	0.3299	0.691873
d.CF_WBI.UHF_WBI	-1.310288	-0.23538	0.32211	0.9247	2.064238

sd.d 0.007798 0.06654 0.13876 0.2479 0.559051

-- Model fit (residual deviance):

Dbar	pD	DIC
62.39525	38.91410	101.30936

62 data points, ratio 1.006, I^2 = 2%

Inconsistency:

Results on the Log Odds Ratio scale

Iterations = 20001:70000

Thinning interval = 1

Number of chains = 4

Sample size per chain = 50000

1. Empirical mean and standard deviation for each variable,

plus standard error of the mean:

	Mean	SD	Naive SE	Time-series SE
d.CF_WBI.HF_WBI	-0.255683	0.1444	0.0003229	0.002229
d.CF_WBI.IMRT	-0.008563	0.4492	0.0010044	0.007978
d.CF_WBI.IOeRT	1.435417	0.4240	0.0009481	0.008040
d.CF_WBI.IORT	0.566100	0.2522	0.0005639	0.003532
d.CF_WBI.ISBT	0.133594	0.2932	0.0006555	0.005746
d.CF_WBI.UHF_WBI	0.304469	0.8529	0.0019071	0.019248
d.HF_WBI.IMRT	0.602652	1.0923	0.0024425	0.030839
sd.d	0.179657	0.1490	0.0003331	0.004162

2. Quantiles for each variable:

	2.5%	25%	50%	75%	97.5%
d.CF_WBI.HF_WBI	-0.575269	-0.33813	-0.244819	-0.1601	-0.003216

```
d.CF_WBI.IMRT    -0.902618 -0.30806 -0.003243  0.2921  0.870512

d.CF_WBI.IOeRT    0.588874  1.16389  1.437817  1.7055  2.274623

d.CF_WBI.IORT     0.061486  0.40588  0.567720  0.7304  1.057003

d.CF_WBI.ISBT    -0.445119 -0.06144  0.133681  0.3295  0.706732

d.CF_WBI.UHF_WBI -1.323147 -0.26123  0.282986  0.8427  2.034009

d.HF_WBI.IMRT    -1.383129 -0.12586  0.539550  1.2494  2.931834

sd.d              0.007272  0.06534  0.142222  0.2548  0.550344



-- Model fit (residual deviance):



    Dbar        pD       DIC

 63.23583  39.88848 103.12432



62 data points, ratio 1.02, I^2 = 4%
```

LR

Consistency:

Results on the Log Odds Ratio scale

Iterations = 20001:70000

Thinning interval = 1

Number of chains = 4

Sample size per chain = 50000

1. Empirical mean and standard deviation for each variable,

plus standard error of the mean:

	Mean	SD	Naive SE	Time-series SE
d.CF_WBI.HF_WBI	-0.18570	0.7175	0.0016045	0.002355
d.CF_WBI.IMRT	0.06169	0.9389	0.0020994	0.003771
d.CF_WBI.IOeRT	0.94405	0.8025	0.0017945	0.002936
d.CF_WBI.IORT	0.44738	0.7918	0.0017705	0.002261
d.CF_WBI.ISBT	0.18630	1.3028	0.0029131	0.006986

sd.d 0.98825 0.4035 0.0009024 0.003045

2. Quantiles for each variable:

	2.5%	25%	50%	75%	97.5%
d.CF_WBI.HF_WBI	-1.6795	-0.598285	-0.18075	0.2323	1.280
d.CF_WBI.IMRT	-1.8349	-0.505613	0.06011	0.6281	1.972
d.CF_WBI.IOeRT	-0.6939	0.476634	0.93843	1.4102	2.601
d.CF_WBI.IORT	-1.1784	-0.009421	0.44503	0.9025	2.076
d.CF_WBI.ISBT	-2.4031	-0.626620	0.17266	0.9899	2.830
sd.d	0.3481	0.680037	0.92980	1.2565	1.862

-- Model fit (residual deviance):

Dbar	pD	DIC
18.34321	17.62632	35.96953

18 data points, ratio 1.019, I^2 = 7%

Inconsistency:

Results on the Log Odds Ratio scale

Iterations = 20001:70000

Thinning interval = 1

Number of chains = 4

Sample size per chain = 50000

1. Empirical mean and standard deviation for each variable,

plus standard error of the mean:

	Mean	SD	Naive SE	Time-series SE
d.CF_WBI.HF_WBI	-0.0387	0.8511	0.0019031	0.002288
d.CF_WBI.IMRT	-0.2739	1.2763	0.0028538	0.004139
d.CF_WBI.IOeRT	0.9546	0.8623	0.0019282	0.003026

d.CF_WBI.IORT   0.4489 0.8556 0.0019131   0.002381

d.CF_WBI.ISBT   0.1965 1.3798 0.0030853   0.006908

d.HF_WBI.IMRT   0.7064 1.4066 0.0031452   0.006749

sd.d   1.0789 0.4203 0.0009397   0.003076

2. Quantiles for each variable:

	2.5%	25%	50%	75%	97.5%
d.CF_WBI.HF_WBI	-1.7888	-0.52969	-0.04211	0.4529	1.721
d.CF_WBI.IMRT	-2.8844	-1.04015	-0.27333	0.4910	2.336
d.CF_WBI.IOeRT	-0.8006	0.44836	0.94847	1.4549	2.741
d.CF_WBI.IORT	-1.3091	-0.04887	0.44588	0.9419	2.219
d.CF_WBI.ISBT	-2.5418	-0.66715	0.18038	1.0454	3.023
d.HF_WBI.IMRT	-2.0920	-0.17970	0.69402	1.5784	3.568
sd.d	0.3826	0.74989	1.03333	1.3886	1.908

-- Model fit (residual deviance):

Dbar pD DIC

18.51486 18.05181 36.56667

18 data points, ratio 1.029, I^2 = 8%

OS

Consistency：

Results on the Log Odds Ratio scale

Iterations = 20001:70000

Thinning interval = 1

Number of chains = 4

Sample size per chain = 50000

1. Empirical mean and standard deviation for each variable,

plus standard error of the mean:

	Mean	SD	Naive SE	Time-series SE
d.CF_WBI.HF_WBI	0.001565	0.1248	0.0002791	0.001463
d.CF_WBI.IMRT	-0.332219	0.3370	0.0007535	0.005006
d.CF_WBI.IOeRT	0.107734	0.3276	0.0007325	0.003271
d.CF_WBI.IORT	-0.199480	0.1708	0.0003819	0.001480
d.CF_WBI.ISBT	-0.088706	0.2739	0.0006124	0.004115
d.CF_WBI.UHF_WBI	0.093797	0.3390	0.0007581	0.003542
sd.d	0.156793	0.1347	0.0003012	0.003305

2. Quantiles for each variable:

	2.5%	25%	50%	75%	97.5%
d.CF_WBI.HF_WBI	-0.248341	-0.07463	0.001888	0.07790	0.2474
d.CF_WBI.IMRT	-1.027086	-0.54427	-0.320398	-0.10991	0.2932
d.CF_WBI.IOeRT	-0.535020	-0.09596	0.106159	0.31113	0.7541
d.CF_WBI.IORT	-0.538116	-0.29895	-0.201545	-0.09994	0.1447

d.CF_WBI.ISBT   -0.612134 -0.26834 -0.094979 0.08383 0.4765

d.CF_WBI.UHF_WBI -0.573929 -0.11823 0.096356 0.30445 0.7612

sd.d   0.007788 0.05809 0.120796 0.21613 0.5092

-- Model fit (residual deviance):

Dbar	pD	DIC
40.83055	28.65744	69.48800

44 data points, ratio 0.928, I^2 = 0%

Inconsistency:

Results on the Log Odds Ratio scale

Iterations = 20001:70000

Thinning interval = 1

Number of chains = 4

Sample size per chain = 50000

1. Empirical mean and standard deviation for each variable,

plus standard error of the mean:

	Mean	SD	Naive SE	Time-series SE
d.CF_WBI.HF_WBI	-0.0003462	0.1266	0.0002832	0.001478
d.CF_WBI.IMRT	-0.3344925	0.3366	0.0007526	0.005132
d.CF_WBI.IOeRT	0.1010957	0.3286	0.0007349	0.003404
d.CF_WBI.IORT	-0.2002646	0.1727	0.0003861	0.001660
d.CF_WBI.ISBT	-0.0855151	0.2794	0.0006247	0.004386
d.CF_WBI.UHF_WBI	0.0970140	0.3438	0.0007687	0.003919
sd.d	0.1581588	0.1376	0.0003077	0.003375

2. Quantiles for each variable:

	2.5%	25%	50%	75%	97.5%

d.CF_WBI.HF_WBI -0.260964 -0.07431 -0.0003423 0.07600 0.2484

d.CF_WBI.IMRT   -1.045492 -0.54150 -0.3201879 -0.10646 0.2809

d.CF_WBI.IOeRT   -0.544350 -0.10289 0.0988478 0.30831 0.7472

d.CF_WBI.IORT   -0.537930 -0.30329 -0.2014145 -0.10026 0.1518

d.CF_WBI.ISBT   -0.607291 -0.27353 -0.0935892 0.09503 0.4792

d.CF_WBI.UHF_WBI -0.569875 -0.12042 0.0932805 0.31356 0.7727

sd.d   0.005892 0.05563 0.1211977 0.22198 0.5150

-- Model fit (residual deviance):

Dbar   pD   DIC

40.90053 28.81158 69.71211

44 data points, ratio 0.9296, I^2 = 0%

DFS

Consistency：

Results on the Log Odds Ratio scale

Iterations = 20001:70000

Thinning interval = 1

Number of chains = 4

Sample size per chain = 50000

1. Empirical mean and standard deviation for each variable,

plus standard error of the mean:

	Mean	SD	Naive SE	Time-series SE
d.CF_WBI.HF_WBI	-0.3604	0.4545	0.001016	0.003125
d.CF_WBI.ISBT	-0.2756	0.7728	0.001728	0.003338
sd.d	0.8088	0.4997	0.001117	0.006346

2. Quantiles for each variable:

	2.5%	25%	50%	75%	97.5%
d.CF_WBI.HF_WBI	-1.4472	-0.5903	-0.2849	-0.06513	0.3676
d.CF_WBI.ISBT	-1.8590	-0.7089	-0.2785	0.15364	1.3205
sd.d	0.0471	0.4377	0.7357	1.10282	1.9999

-- Model fit (residual deviance):

Dbar	pD	DIC
21.32961	15.90420	37.23381

18 data points, ratio 1.185, I^2 = 20%

Inconsistency:

Results on the Log Odds Ratio scale

Iterations = 20001:70000

Thinning interval = 1

Number of chains = 4

Sample size per chain = 50000

1. Empirical mean and standard deviation for each variable,

plus standard error of the mean:

	Mean	SD	Naive SE	Time-series SE
d.CF_WBI.HF_WBI	-0.3564	0.4524	0.001012	0.003027
d.CF_WBI.ISBT	-0.2765	0.7699	0.001722	0.003399
sd.d	0.8054	0.5026	0.001124	0.006306

2. Quantiles for each variable:

	2.5%	25%	50%	75%	97.5%
d.CF_WBI.HF_WBI	-1.44296	-0.5863	-0.2804	-0.06357	0.3655
d.CF_WBI.ISBT	-1.86214	-0.7051	-0.2784	0.15183	1.3170
sd.d	0.05425	0.4310	0.7283	1.09861	2.0084

-- Model fit (residual deviance):

    Dbar   pD   DIC

21.37549 15.89257 37.26806

18 data points, ratio 1.188, I^2 = 20%

Cosmesis

Consistency：

Results on the Log Odds Ratio scale

Iterations = 20001:70000

Thinning interval = 1

Number of chains = 4

Sample size per chain = 50000

1. Empirical mean and standard deviation for each variable,

plus standard error of the mean:

	Mean	SD	Naive SE	Time-series SE
d.CF_WBI.3D_CRT	-2.2510	1.1221	0.0025090	0.009718
d.CF_WBI.Balloon_catheter	0.5366	0.9074	0.0020291	0.003056
d.CF_WBI.HF_WBI	-0.1378	0.4350	0.0009726	0.003079
d.CF_WBI.IMRT	-2.1920	1.1214	0.0025075	0.012279
d.CF_WBI.IOeRT	-1.4929	0.7433	0.0016620	0.004837
d.CF_WBI.ISBT	-0.7535	0.5298	0.0011848	0.001792
sd.d	0.7080	0.4423	0.0009891	0.006534

2. Quantiles for each variable:

	2.5%	25%	50%	75%	97.5%
d.CF_WBI.3D_CRT	-4.66098	-2.8832	-2.19070	-1.5670	-0.1611
d.CF_WBI.Balloon_catheter	-1.32731	0.0553	0.53610	1.0101	2.4009

d.CF_WBI.HF_WBI -1.16523 -0.3495 -0.07831 0.1285 0.5885

d.CF_WBI.IMRT -4.72408 -2.8332 -2.07941 -1.4232 -0.3275

d.CF_WBI.IOeRT -3.20843 -1.8702 -1.40326 -1.0309 -0.2494

d.CF_WBI.ISBT -1.87320 -1.0255 -0.73608 -0.4725 0.3064

sd.d 0.05591 0.4007 0.63390 0.9276 1.8184

-- Model fit (residual deviance):

Dbar pD DIC

32.47655 26.51983 58.99638

32 data points, ratio 1.015, I^2 = 5%

Inconsistency：

Results on the Log Odds Ratio scale

Iterations = 20001:70000

Thinning interval = 1

Number of chains = 4

Sample size per chain = 50000

1. Empirical mean and standard deviation for each variable,

plus standard error of the mean:

	Mean	SD	Naive SE	Time-series SE
d.3D_CRT.CF_WBI	-11.1433	33.4447	0.0747846	5.736355
d.3D_CRT.HF_WBI	2.1140	0.9640	0.0021555	0.006919
d.Balloon_catheter.CF_WBI	-0.5519	0.8304	0.0018567	0.003006
d.CF_WBI.HF_WBI	-0.0108	0.3955	0.0008843	0.001966
d.CF_WBI.IMRT	-32.8733	19.7382	0.0441360	3.119481
d.CF_WBI.IOeRT	-1.4471	0.6904	0.0015438	0.004702
d.CF_WBI.ISBT	-0.7461	0.4859	0.0010866	0.001791
d.HF_WBI.IMRT	-0.7605	1.2363	0.0027643	0.010268
sd.d	0.6322	0.4084	0.0009132	0.005963

2. Quantiles for each variable:

	2.5%	25%	50%	75%	97.5%
d.3D_CRT.CF_WBI	-86.92417	-33.5675	-9.88338	9.64496	57.4779
d.3D_CRT.HF_WBI	0.23122	1.5407	2.09025	2.67306	4.0882
d.Balloon_catheter.CF_WBI	-2.24915	-0.9942	-0.55181	-0.10857	1.1492
d.CF_WBI.HF_WBI	-0.89975	-0.2063	0.02471	0.21390	0.7181
d.CF_WBI.IMRT	-76.81844	-46.2522	-31.40557	-16.64129	-3.5340
d.CF_WBI.IOeRT	-3.04165	-1.8002	-1.36836	-1.00674	-0.2956
d.CF_WBI.ISBT	-1.77880	-0.9974	-0.72740	-0.48278	0.2129
d.HF_WBI.IMRT	-3.32438	-1.5202	-0.72550	0.03425	1.6068
sd.d	0.04317	0.3472	0.56539	0.83660	1.6440

-- Model fit (residual deviance):

Dbar pD DIC

30.32381 26.01635 56.34015

32 data points, ratio 0.9476, I^2 = 0%

---

2 Supplementary Table

Supplementary Table 1 Summary Table of Adverse Events

Outcome	Study	Treatment	n	t	Incidence rate (%)
Acute AE Total	Icro Meattini (2020)	IMRT	246	52	21.1%
		CF-WBI	260	173	66.5%
	Budhi Singh Yadav (2020)	3D-CRT	65	21	32.3%
		HF-WBI	67	29	43.3%
	Francesca De Felice (2017)	CF-WBI	62	62	100.0%
		HF-WBI	58	58	100.0%
Total Acute AE (⩾G2)	Icro Meattini (2020)	IMRT	246	5	2.0%
		CF-WBI	260	98	37.7%
	Simona F. Shaitelman (2018)	CF-WB	149	116	77.9%
		HF-WBI	138	65	47.1%
	Budhi Singh Yadav (2020)	3D-CRT	65	5	7.7%
		HF-WBI	67	10	14.9%
Late AE Total	Icro Meattini (2020)	IMRT	246	11	4.5%
		CF-WBI	260	78	30.0%
	Núria Rodríguez (2013)	CF-WBI	51	8	15.7%
		3D-CRT	51	6	11.8%
	Adrian Murray Brunt (2020)	CF-WBI	986	98	9.9%
		HF-WBI	1005	155	15.4%
	Letizia Deantonio (2016)	HF-WBI	85	4	4.7%
		CF-WBI	70	4	5.7%
	Francesca De Felice (2017)	CF-WBI	62	17	27.4%
		HF-WBI	58	11	19.0%
Total Late AE (⩾G2)	Icro Meattini (2020)	IMRT	246	0	0.0%
		CF-WBI	260	7	2.7%
	Simona F. Shaitelman (2018)	CF-WBI	142	46	32.4%
		HF-WBI	129	40	31.0%
	Núria Rodríguez (2013)	CF-WBI	51	0	0.0%
		3D-CRT	51	0	0.0%
Acute Skin Toxicity	Lorenzo Livi (2015)	CF-WBI	260	173	66.5%
		IMRT	246	49	19.9%
	Kumiko Karasawa (2014)	HF-WBI	717	55	77.1%
		CF-WBI	381	3	94.8%

				361	
	Rajiv K Agrawal (2011)	CF-WB<br>HF-WBI	302<br>308	102<br>83	33.8%<br>26.9%
	Csaba Polgár (2004)	Interstitial Brachytherapy<br>CF-WBI	45<br>35	6<br>4	13.3%<br>11.4%
	Shu-Lian Wang (2020)	CF-WBI<br>HF-WBI	363<br>365	340<br>329	93.7%<br>90.1%
	Davide Franceschini (2021)	HF-WBI<br>IMRT	90<br>82	56<br>11	62.2%<br>13.4%
Late Skin Toxicity	Vratislav Strnad (2016)	Interstitial Brachytherapy<br>CF-WBI	527<br>442	17<br>25	3.2%<br>5.7%
	Lorenzo Livi (2015)	CF-WB<br>IMRT	260<br>246	29<br>11	11.2%<br>4.5%
	Umberto Veronesi (2013)	CF-WBI<br>IOeRT	412<br>464	5<br>6	1.2%<br>1.3%
	Csaba Polgár (2017)	Interstitial Brachytherapy<br>CF-WBI	484<br>393	69<br>69	14.3%<br>17.6%
	Timothy J. Whelan (2010)	CF-WBI<br>HF-WBI	220<br>235	65<br>78	29.5%<br>33.2%
	Davide Franceschini (2021)	HF-WBI<br>IMRT	90<br>82	37<br>15	41.1%<br>18.3%
	Timothy Whelan (2002)	HF-WBI<br>CF-WBI	394<br>358	13<br>18	3.3%<br>5.0%
Subcutaneous tissue effect	Vratislav Strnad (2016)	Interstitial Brachytherapy<br>CF-WBI	527<br>442	40<br>28	7.6%<br>6.3%
	Csaba Polgár (2017)	Interstitial Brachytherapy<br>CF-WBI	485<br>393	204<br>145	42.1%<br>36.9%
	Timothy J. Whelan (2010)	CF-WBI<br>HF-WBI	220<br>235	120 	54.5%<br>51.9%

				12<br>2	
	Timothy Whelan (2002)	HF-WBI<br>CF-WBI	394<br>358	34<br>40	8.6%<br>11.2%
Fibrosis	Vratislav Strnad (2016)	Interstitial<br>Brachytherap<br>y<br>CF-WBI	527<br>442	0<br>1	0.0%<br>0.2%
	Budhi Singh Yadav (2020)	3D-CRT<br>HF-WBI	65<br>67	15<br>28	23.1%<br>41.8%
	Csaba Polgár (2004)	Interstitial<br>Brachytherap<br>y<br>CF-WBI	45<br>35	20<br>10	44.4%<br>28.6%
	Shu-Lian Wang (2020)	CF-WBI<br>HF-WBI	363<br>365	32<br>27	8.8%<br>7.4%
	Csaba Polgár (2017)	Interstitial<br>Brachytherap<br>y<br>CF-WBI	484<br>392	18<br>7<br>13<br>8	38.6%<br>5.2%
Late Pain	Vratislav Strnad (2016)	Interstitial<br>Brachytherap<br>y<br>CF-WBI	527<br>442	6<br>14	1.1%<br>3.2%
	Jessica L. Wobb (2015)	Balloon<br>catheter<br>CF-WBI	327<br>489	12<br>10	3.7%<br>2.0%
	Csaba Polgár (2017)	Interstitial<br>Brachytherap<br>y<br>CF-WBI	484<br>393	10<br>5<br>84	21.7%<br>21.4%
Late Edema	Sea-Won Lee (2016)	HF-WBI<br>CF-WBI	258<br>278	0<br>3	0.0%<br>1.1%
	Jessica L. Wobb (2015)	Balloon<br>catheter<br>CF-WBI	327<br>489	4<br>17	1.2%<br>3.5%
	Budhi Singh Yadav (2020)	3D-CRT<br>HF-WBI	65<br>67	5<br>10	7.7%<br>14.9%
	Shu-Lian Wang (2020)	CF-WBI<br>HF-WBI	363<br>365	40<br>34	11.0%<br>9.3%
	Csaba Polgár (2017)	Interstitial<br>Brachytherap<br>y<br>CF-WBI	483<br>393	11<br>16	2.3%<br>4.1%

Late Hyperpigmentation	Jessica L. Wobb (2015)	Balloon catheter<br>CF-WBI	327<br>489	9<br>56	2.8%<br>11.5%
	Budhi Singh Yadav (2020)	3D-CRT<br>HF-WBI	65<br>67	6<br>15	9.2%<br>22.4%
	Csaba Polgár (2017)	Interstitial Brachytherapy<br>CF-WBI	484<br>392	27<br>40	5.6%<br>10.2%
Late Fat Necrosis	Jessica L. Wobb (2015)	Balloon catheter<br>CF-WBI	327<br>489	19<br>4	5.8%<br>0.8%
	Csaba Polgár (2004)	Interstitial Brachytherapy<br>CF-WBI	45<br>35	10<br>6	22.2%<br>17.1%
	Antonella Ciabattoni (2021)	IOeRT<br>CF-WBI	125<br>110	2<br>0	1.6%<br>0.0%
	Csaba Polgár (2017)	Interstitial Brachytherapy<br>CF-WBI	484<br>393	44<br>27	9.1%<br>6.9%

Abbreviations: CF-WBI, conventional fractionated whole-breast irradiation; HF-WBI, hypofractionated whole-breast irradiation; UHF-WBI, ultra-hypofractionated whole-breast irradiation; IORT, intraoperative radiotherapy; IOeRT, intraoperative electron radiotherapy; IMRT, intensity-modulated radiation therapy; ISBT, interstitial brachytherapy; 3D-CRT, three-dimensional conformal radiotherapy; IBR, ipsilateral breast recurrence; LR, locoregional recurrence; OS, overall survival; DFS, disease-free survival

3 Supplementary Figures

Supplementary Figure 1

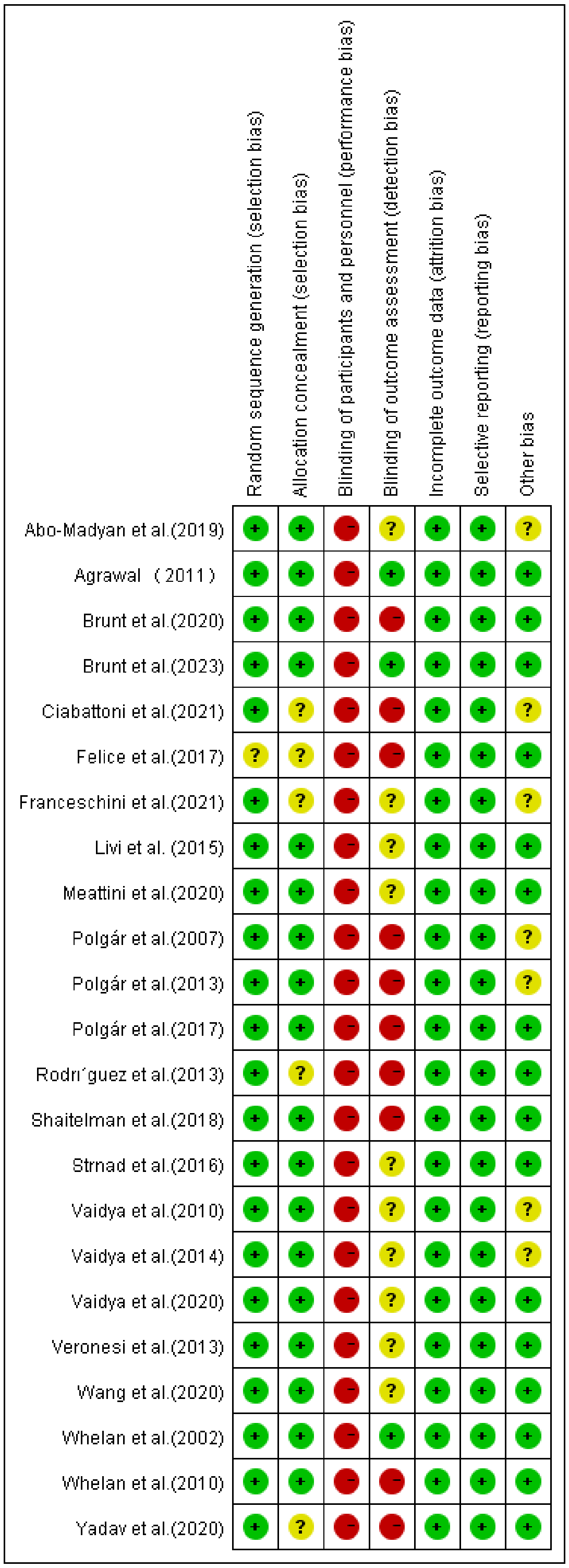

Figure 1. Risk of bias summary

Supplementary Figure 2

a

					ISBT
				IORT	0.77 (0.04, 16.9)
			IOeRT	0.61 (0.06, 6.27)	0.47 (0.02, 10.51)
		IMRT	2.42 (0.19, 30.28)	1.47 (0.12, 17.94)	1.13 (0.04, 28.98)
	HF-WBI	1.27 (0.19, 9.24)	3.06 (0.35, 28.77)	1.86 (0.21, 17.46)	1.43 (0.07, 30.05)
CF-WBI	0.83 (0.19, 3.6)	1.06 (0.16, 7.18)	2.56 (0.5, 13.47)	1.56 (0.31, 7.97)	1.19 (0.09, 16.94)

b

						UHF-WBI
					ISBT	1.21 (0.5, 2.76)
				IORT	1.11 (0.6, 2.15)	1.35 (0.63, 2.83)
			IOeRT	0.74 (0.36, 1.52)	0.82 (0.37, 1.94)	0.99 (0.39, 2.51)
		IMRT	1.53 (0.64, 4.09)	1.13 (0.57, 2.5)	1.26 (0.57, 3.21)	1.51 (0.62, 4.09)
	HF-WBI	0.73 (0.34, 1.39)	1.11 (0.56, 2.24)	0.82 (0.54, 1.25)	0.91 (0.52, 1.7)	1.1 (0.54, 2.25)
CF-WBI	1 (0.78, 1.28)	0.73 (0.36, 1.34)	1.11 (0.59, 2.13)	0.82 (0.58, 1.16)	0.91 (0.54, 1.61)	1.1 (0.56, 2.14)

c

		ISBT
	HF-WBI	1.01 (0.21, 8.12)
CF-WBI	0.75 (0.24, 1.44)	0.76 (0.16, 3.75)

Figure 2. Results of the network meta-analysis for (a) locoregional recurrence, (b) overall survival, and (c) disease-free survival. CrI, credible interval; CF-WBI, conventional fractionated whole-breast irradiation; HF-WBI, hypofractionated whole-breast irradiation; UHF-WBI, ultra-hypofractionated whole-breast irradiation; IORT, intraoperative radiotherapy; IOeRT, intraoperative electron radiotherapy; IMRT, intensity-modulated radiation therapy; ISBT, interstitial brachytherapy; 3D-CRT, three-dimensional conformal radiotherapy. Each cell represents the comparison (HR and 95% CrI) between the row treatment and the column treatment.

Supplementary Figure 3

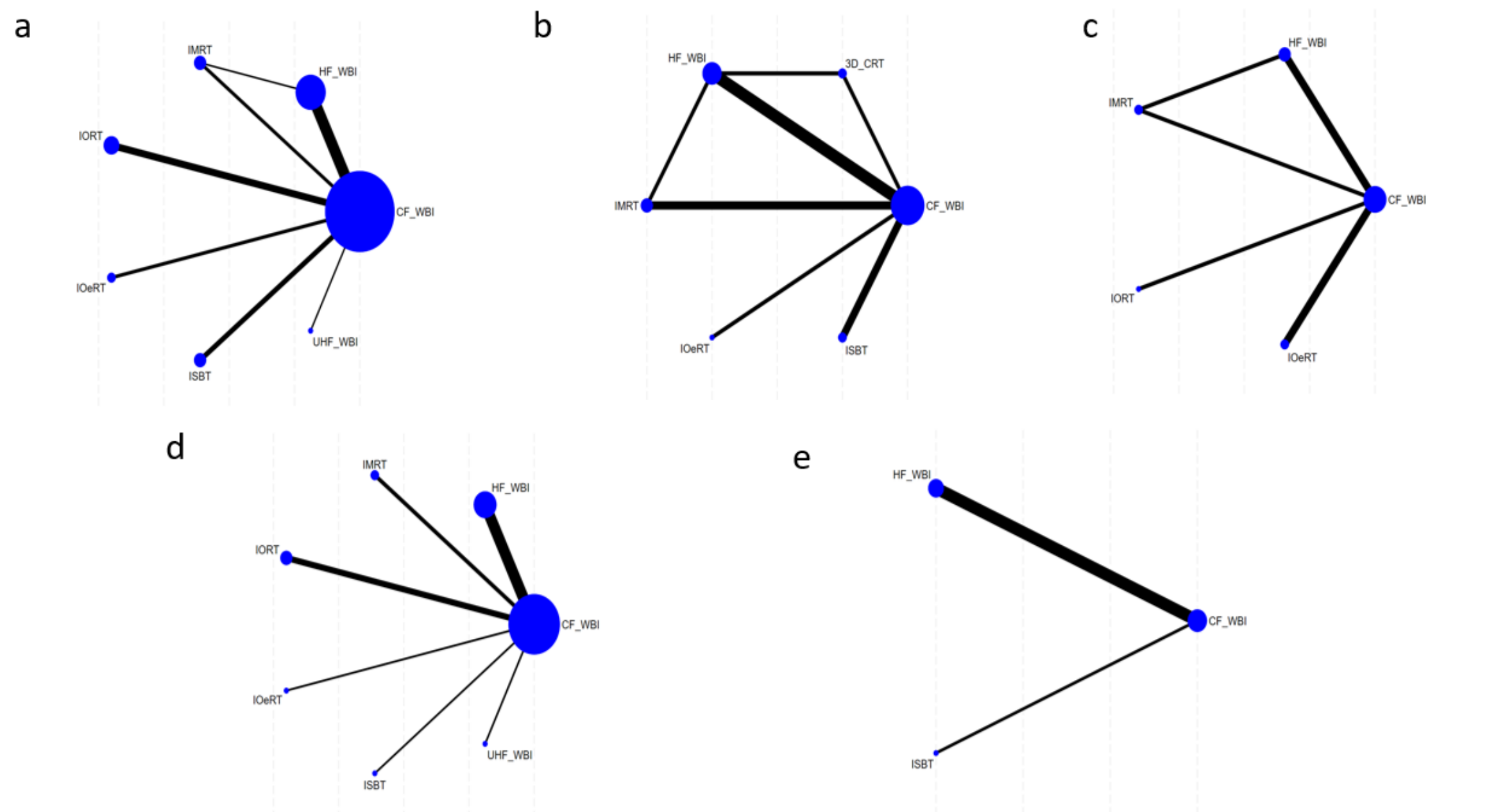

Figure 1. RCT-only network of treatment comparisons for (a) ipsilateral breast recurrence, (b) cosmetic outcomes, (c) locoregional recurrence, (d) overall survival, and (e) disease-free survival. CF-WBI, conventional fractionated whole-breast irradiation; HF-WBI, hypofractionated whole-breast irradiation; UHF-WBI, ultra-hypofractionated whole-breast irradiation; IORT, intraoperative radiotherapy; IOeRT, intraoperative electron radiotherapy; IMRT, intensity-modulated radiation therapy; ISBT, interstitial brachytherapy; 3D-CRT, three-dimensional conformal radiotherapy.

Supplementary Figure 4

a

						UHF-WBI
					ISBT	0.95 (0.14, 6.65)
				IORT	0.82 (0.32, 2.3)	0.78 (0.13, 5.21)
			IOeRT	0.42 (0.15, 1.36)	0.35 (0.11, 1.31)	0.33 (0.05, 2.65)
		IMRT	**3.98 (1.01, 13.53)**	1.68 (0.57, 4.77)	1.38 (0.42, 4.61)	1.3 (0.19, 9.65)
	HF-WBI	1.28 (0.52, 3.48)	**5.08 (1.8, 13.74)**	**2.13 (1.07, 4.74)**	1.75 (0.75, 4.76)	1.67 (0.28, 11.14)
CF-WBI	0.82 (0.47, 1.2)	1.04 (0.43, 2.56)	**4.15 (1.49, 10.21)**	1.75 (0.94, 3.09)	1.43 (0.66, 3.28)	1.35 (0.23, 8.21)

b

					ISBT
				IOeRT	0.94 (0.13, 5.56)
			IMRT	3.52 (0.45, 72.36)	3.24 (0.52, 48.97)
		HF-WBI	**0.14 (0.01, 0.7)**	0.48 (0.1, 3.4)	0.44 (0.13, 1.99)
	CF-WBI	1.13 (0.39, 2.15)	**0.15 (0.01, 0.73)**	0.54 (0.12, 2.46)	0.5 (0.16, 1.34)
3D-CRT	**7.23 (1.28, 66.92)**	**8.07 (1.47, 53.37)**	1.1 (0.06, 12.44)	3.87 (0.41, 59.92)	3.62 (0.46, 40.59)

c

				IORT
			IOeRT	0.57 (0.02, 12.73)
		IMRT	2.43 (0.15, 40.51)	1.39 (0.05, 36.29)
	HF-WBI	1.28 (0.15, 11.34)	3.11 (0.27, 38.91)	1.77 (0.09, 36.71)
CF-WBI	0.83 (0.16, 4.21)	1.06 (0.13, 8.76)	2.59 (0.42, 16.76)	1.47 (0.12, 18.34)

d

						UHF-WBI
					ISBT	1.53 (0.58, 4.03)
				IORT	0.87 (0.4, 1.9)	1.34 (0.62, 2.89)
			IOeRT	0.74 (0.35, 1.6)	0.65 (0.25, 1.69)	0.99 (0.38, 2.6)
		IMRT	1.53 (0.62, 4.2)	1.13 (0.56, 2.65)	0.99 (0.4, 2.79)	1.52 (0.6, 4.26)
	HF-WBI	0.71 (0.32, 1.42)	1.1 (0.52, 2.26)	0.81 (0.52, 1.3)	0.71 (0.34, 1.52)	1.08 (0.52, 2.31)
CF-WBI	1.01 (0.76, 1.34)	0.72 (0.34, 1.36)	1.11 (0.56, 2.15)	0.82 (0.58, 1.19)	0.72 (0.36, 1.44)	1.1 (0.56, 2.21)

e

		IMRT
	HF-WBI	1.04 (0.33, 3.13)
CF-WBI	1.05 (0.83, 1.34)	1.09 (0.36, 3.19)

Figure 4. Results of the RCT-onllu network meta-analysis for (a) ipsilateral breast recurrence, (b) cosmesis,(c) locoregional recurrence, (d) overall survival, and (e) disease-free survival. CrI, credible interval; CF-WBI, conventional fractionated whole-breast irradiation; HF-WBI, hypofractionated whole-breast irradiation; UHF-WBI, ultra-hypofractionated whole-breast irradiation; IORT, intraoperative radiotherapy; IOeRT, intraoperative electron radiotherapy; IMRT, intensity-modulated radiation therapy; ISBT, interstitial brachytherapy; 3D-CRT, three-dimensional conformal radiotherapy. Each cell represents the comparison (HR and 95% CrI) between the row treatment and the column treatment. All bolded values are statistically significant at p = 0.05.
